# Epigallocatechin Gallate Modulates the Cellular Response to Doxorubicin in HeLa Cervical Cancer Cells

**DOI:** 10.3390/biom16091299

**Published:** 2026-09-08

**Authors:** Mehmet Emin Ayağ, Mehmet Cudi Tuncer, Şamil Öztürk

**Affiliations:** 1 Department of Gynecology and Obstetrics, Mehmet Emin Ayağ Practice, Mardin 47000, Turkey; 2 Department of Anatomy, Faculty of Medicine, Dicle University, Diyarbakır 21280, Turkey; drcudi@hotmail.com; 3 Vocational School of Health Services, Çanakkale Onsekiz Mart University, Çanakkale 17100, Turkey; ozturksamil@outlook.com

**Keywords:** epigallocatechin gallate, doxorubicin, cervical cancer, HeLa, apoptosis, *EGFR*, ROS

## Abstract

Cervical cancer remains a major cause of cancer-related mortality worldwide, highlighting the need for strategies that may improve responses to conventional chemotherapeutics. Epigallocatechin gallate (EGCG), a green-tea polyphenol with diverse biological activities, has been investigated as a potential chemosensitizing agent. This study evaluated the interaction between EGCG and doxorubicin (DOX) in HeLa cervical cancer cells, with HaCaT keratinocytes included as a non-malignant comparator. Cell viability and drug interactions were assessed using the CCK-8 assay and Chou–Talalay combination index (CI) analysis. Complementary assays evaluated membrane integrity, wound closure, apoptosis, cell-cycle distribution, intracellular DCF-associated fluorescence with or without N-acetylcysteine (NAC) pretreatment, caspase-3 immunoreactivity, and *EGFR*, *FOXP3*, *CASP3*, and *CASP7* mRNA expression. Network-based analyses were additionally used to identify candidate molecular associations and pathways. CI analysis demonstrated synergistic EGCG–DOX interactions in HeLa cells under the tested conditions, whereas additive or antagonistic interactions predominated in HaCaT cells. Combined treatment produced the greatest reduction in viable cells, increased apoptosis, altered cell-cycle distribution, and reduced wound closure. It also produced the highest viability-normalized DCF-associated fluorescence, which was attenuated by NAC pretreatment, indicating an antioxidant-sensitive change in intracellular oxidative status without establishing a causal role in cytotoxicity. Combined treatment was further associated with increased total caspase-3 immunoreactivity and altered *EGFR*, *FOXP3*, *CASP3*, and *CASP7* transcript levels. Overall, EGCG and DOX exhibited synergistic interactions and multiple treatment-associated cellular and transcriptional responses in HeLa cells under the present in vitro conditions. These findings do not establish cancer-specific selectivity or a definitive molecular mechanism but provide a basis for validation in additional cervical cancer models and at clinically relevant exposures.

## 1. Introduction

Cervical cancer is the fourth most common malignancy among women worldwide and remains a significant global health problem, ranking as the fourth leading cause of cancer-related death in women [1]. The disease burden is disproportionately concentrated in low- and middle-income countries, where approximately 85% of new cases and 90% of deaths occur, partly because limited access to screening programs and healthcare infrastructure contributes to delayed diagnosis and poorer clinical outcomes [2]. Persistent infection with high-risk human papillomavirus (HPV) types, particularly HPV-16 and HPV-18, is the major etiological factor in cervical cancer [3]. Although prophylactic HPV vaccination and screening are central to prevention, effective treatment remains necessary for patients with established disease [4]. According to current European guidelines, treatment is stage- and risk-adapted and includes surgery, radiotherapy, concurrent chemoradiotherapy, and systemic treatment for recurrent or metastatic disease [5]. Despite advances in treatment, therapeutic resistance and treatment-related toxicities remain important challenges, supporting continued investigation of combination strategies that may improve treatment responses [6,7,8,9,10,11].

EGCG is the most abundant catechin derivative in green tea (*Camellia sinensis*) and has been widely investigated for its antioxidant, anti-inflammatory, and tumor cell-modulating properties. Studies in HeLa cervical cancer cells have shown that EGCG can suppress cell proliferation and induce apoptosis, with reported involvement of NF-κB- and Akt-related signaling [12]. EGCG has also been reported to induce redox-dependent inactivation of thioredoxin (Trx) and thioredoxin reductase (TrxR), accompanied by increased intracellular ROS levels in HeLa cells [13]. These observations illustrate the context-dependent biological effects of EGCG, which may exhibit antioxidant or pro-oxidant properties depending on the experimental setting. Furthermore, EGCG has been reported to induce ROS-associated lysosomal membrane permeabilization and cell death in experimental cancer-cell models [14,15]. In contrast, antioxidant-related effects of EGCG have also been described in other experimental contexts [16]. However, the translational applicability of EGCG is constrained by gastrointestinal instability, extensive metabolism, and low oral bioavailability [17].

The combination of EGCG with DOX has been investigated in several experimental cancer models. Synergistic or enhanced treatment responses have been reported in bladder cancer, hepatocellular carcinoma, and osteosarcoma cells, with proposed associations involving NF-κB/MDM2/p53 signaling, autophagy, and cancer stem-cell characteristics [15,18,19]. EGCG has also been reported to modulate multidrug-resistance-related processes and DOX-associated cardiotoxicity in experimental models [20]. EGFR is a key regulator of cellular proliferation and survival, and altered EGFR expression has been documented in cervical carcinogenesis [21,22]. FOXP3 has also been investigated as a treatment-responsive transcriptional marker in experimental cancer-cell models, including in the context of polyphenol and DOX exposure [23]. However, despite these observations in other tumor models, the pharmacological interaction between EGCG and DOX has not been sufficiently characterized in cervical cancer cells. In particular, quantitative assessment of EGCG–DOX interactions together with complementary evaluation of apoptosis, cell-cycle responses, intracellular oxidative status, and treatment-associated transcriptional changes remains limited. Moreover, comparative information on the response of HeLa cells and a non-malignant comparator to this combination is scarce. This represents the specific knowledge gap addressed in the present study.

Accordingly, this study investigated the interaction between EGCG and DOX in HeLa cervical cancer cells, with HaCaT keratinocytes included as a non-malignant comparator. We hypothesized that combined exposure would produce a measurable pharmacological interaction and treatment-associated cellular responses rather than presupposing a specific molecular mechanism. Drug interaction was quantitatively evaluated using Chou–Talalay analysis, while complementary cellular, oxidative-status, transcriptional, and network-based analyses were used to characterize the response to treatment. The study was therefore designed to determine whether EGCG and DOX interact synergistically under the tested in vitro conditions and to identify associated biological changes that may provide a basis for subsequent mechanistic validation.

## 2. Materials and Methods

### 2.1. Cell Models, Culture Conditions, and Preparation of Treatment Compounds

HeLa (ATCC^®^ CCL-2™) and HaCaT (CLS Cat. No. 300493) cell lines were obtained from the American Type Culture Collection (ATCC, Manassas, VA, USA) and Cell Lines Service (CLS, Eppelheim, Germany), respectively. Both cell lines were maintained in Dulbecco’s Modified Eagle Medium (DMEM; Gibco, Grand Island, NY, USA) supplemented with 10% fetal bovine serum (FBS; Gibco, Thermo Fisher Scientific, Waltham, MA, USA), 100 U/mL penicillin, and 100 μg/mL streptomycin (Gibco). Cells were cultured at 37 °C in a humidified atmosphere containing 5% CO_2_ and were routinely passaged upon reaching approximately 70–80% confluence. Only cultures displaying normal morphology and appropriate growth characteristics were used for subsequent experiments.

EGCG (EGCG; purity ≥ 95%) and N-acetyl-L-cysteine (NAC; Cat. No. A7250) were obtained from Sigma-Aldrich (St. Louis, MO, USA), whereas DOX was obtained from KOÇAK Pharma (Istanbul, Türkiye). EGCG was dissolved in sterile distilled water to prepare a 100 mM stock solution, sterilized by filtration through a 0.22 μm membrane filter, aliquoted, protected from light, and stored at −20 °C until use. DOX was dissolved in sterile distilled water to prepare a 10 mM stock solution, aliquoted, protected from light, and stored at −20 °C. NAC was prepared in sterile distilled water as a 1 M stock solution and sterilized by filtration through a 0.22 μm membrane filter. Working solutions of EGCG, DOX, and NAC were prepared by dilution in the appropriate culture medium immediately before use. EGCG and DOX aliquots were thawed only once, and repeated freeze–thaw cycles were avoided to minimize potential compound degradation.

### 2.2. Concentration–Response Profiling and Experimental Dose Selection

Cell viability was evaluated using the Cell Counting Kit-8 (CCK-8; Sigma-Aldrich, St. Louis, MO, USA) according to the manufacturer’s instructions. HeLa and HaCaT cells were seeded into 96-well plates at a density of 1 × 10^4^ cells/well and allowed to adhere for 24 h at 37 °C in a humidified atmosphere containing 5% CO_2_. Cells were subsequently exposed to increasing concentrations of EGCG or DOX for 24 or 48 h. Untreated cells maintained under the same culture conditions served as the control group.

At the end of each treatment period, 10 μL of CCK-8 reagent was added to each well, followed by incubation for 2 h at 37 °C. Absorbance was measured at 450 nm using a microplate reader. Cell viability was expressed as a percentage relative to the untreated control, which was defined as 100%. Concentration–response curves were generated, and half-maximal inhibitory concentration (IC_50_) values were estimated by nonlinear regression using a log(inhibitor) versus response model with a variable slope in GraphPad Prism version 8.0 (GraphPad Software, San Diego, CA, USA).

Based on the 48 h concentration–response profiles, EGCG at 100 μM and DOX at 1.5 μM were selected as the working concentrations for subsequent mechanistic experiments. These concentrations were selected as experimentally relevant working doses close to the respective 48 h IC_50_ region while allowing the effects of the individual treatments and their combination to be evaluated under the same experimental conditions. Accordingly, subsequent experiments included untreated control, EGCG (100 μM), DOX (1.5 μM), and EGCG (100 μM) + DOX (1.5 μM) groups unless otherwise specified. The selected EGCG concentration was intended as an experimentally defined in vitro working concentration based on the concentration–response profile and should not be interpreted as representing a clinically or physiologically achievable systemic EGCG exposure.

### 2.3. Fixed-Ratio Drug Interaction Profiling by the Chou–Talalay Method

Drug interactions between EGCG and DOX were evaluated using the Chou–Talalay combination index (CI) method [24]. Following characterization of the individual concentration–response profiles, HeLa and HaCaT cells were exposed to EGCG and DOX using three predefined relative-dose combination schemes, designated 1:1, 1:2, and 2:1. These designations represent relative dose proportions within the combination design and should not be interpreted as direct molar concentration ratios between EGCG and DOX. For the 1:1 scheme, the EGCG/DOX concentration pairs were 12.5/0.125, 25/0.25, 50/0.50, 100/1.00, and 200/2.00 μM. For the 1:2 scheme, the corresponding concentration pairs were 12.5/0.25, 25/0.50, 50/1.00, 100/2.00, and 200/4.00 μM. For the 2:1 scheme, the concentration pairs were 25/0.125, 50/0.25, 100/0.50, 200/1.00, and 400/2.00 μM.

Following treatment, cell viability was determined using the CCK-8 assay as described above. Fraction affected (Fa) values were derived from the treatment-induced reduction in cell viability, with Fa representing the fraction of cells affected by the combination treatment. CI values were calculated using CompuSyn software (ComboSyn Inc., Paramus, NJ, USA) according to the Chou–Talalay method. For each relative-dose combination scheme, the CI value was interpreted at the corresponding experimentally observed Fa level. For interpretation, CI < 0.90 was classified as synergistic, 0.90 ≤ CI ≤ 1.10 as additive, and CI > 1.10 as antagonistic. The analysis was based on three independent experiments. The reported CI values corresponded to the experimentally observed fraction affected (Fa) for each relative-dose combination scheme rather than to a single prespecified Fa level. In HeLa cells, the 1:1, 1:2, and 2:1 relative-dose combination schemes yielded Fa values of 0.68, 0.78, and 0.60, respectively; the corresponding Fa values in HaCaT cells were 0.35, 0.30, and 0.40. Thus, the reported CI values represent interaction estimates at the respective observed effect levels for each tested combination condition.

### 2.4. Fluorescence-Based Assessment of Cell Viability and Membrane Integrity by Calcein-AM/PI Staining

To provide an orthogonal assessment of cell viability and membrane integrity independent of the CCK-8 metabolic readout, HeLa cells were evaluated using dual fluorescence staining with calcein acetoxymethyl ester (Calcein-AM) and propidium iodide (PI). Cells were seeded in 24-well culture plates and allowed to adhere for 24 h under standard culture conditions (37 °C, 5% CO_2_). The cells were subsequently assigned to four experimental groups: untreated control, EGCG (100 μM), DOX (1.5 μM), and EGCG (100 μM) + DOX (1.5 μM). Cells were exposed to the indicated treatments for 48 h.

Following treatment, the culture medium was removed, and the cells were gently washed twice with phosphate-buffered saline (PBS). Calcein-AM and propidium iodide (PI) (Sigma-Aldrich, Merck KGaA, Darmstadt, Germany) were used for fluorescence-based viability assessment. A freshly prepared staining solution containing Calcein-AM at a final concentration of 2 μM and PI at a final concentration of 4 μM in PBS was added to each well at a volume of 500 μL per well. Calcein-AM and PI staining solutions were prepared according to the manufacturer’s instructions and applied to the cells. The cells were incubated with the staining solution for 30 min at 37 °C in the dark. Calcein-AM was used as an indicator of viable cells with preserved intracellular esterase activity and membrane integrity, whereas PI was used to identify cells with compromised plasma membrane integrity. Following incubation, excess staining solution was removed, and the cells were gently washed with PBS before fluorescence imaging.

Fluorescence images were acquired immediately after staining using an inverted fluorescence microscope equipped with appropriate filter sets for the detection of Calcein-associated green fluorescence and PI-associated red fluorescence. All images were acquired under identical imaging conditions, including magnification, exposure time, illumination intensity, camera gain, and acquisition settings, to ensure comparability among experimental groups. Calcein-AM-positive cells were classified as viable cells, whereas PI-positive cells were classified as membrane-compromised/dead cells. Merged images were additionally generated to visualize the relative distribution of viable and membrane-compromised cells within the same microscopic fields.

For quantitative analysis, five randomly selected, non-overlapping microscopic fields were evaluated for each experimental sample. Calcein-AM-positive and PI-positive cells were quantified separately using ImageJ software (version 1.54; National Institutes of Health, Bethesda, MD, USA), applying identical image-analysis criteria across all treatment groups. The percentages of Calcein-AM-positive viable cells and PI-positive dead cells were calculated relative to the total number of cells evaluated within each field. Measurements obtained from the five microscopic fields of the same experimental sample were averaged to generate a single value for that biological replicate; individual microscopic fields were not treated as independent biological observations.

The experiment was performed in three independent biological replicates (*n* = 3). Quantitative results are presented as mean ± standard deviation (SD). Statistical comparisons among the four experimental groups were performed using one-way analysis of variance (ANOVA), followed by Tukey’s multiple-comparisons post hoc test. A two-sided *p* value < 0.05 was considered statistically significant.

### 2.5. Scratch-Wound Assessment of Treatment-Associated Wound Closure

HeLa cells were seeded into 6-well plates at a density of 5 × 10^5^ cells/well and cultured until a confluent monolayer was established. A linear scratch was generated across the cell monolayer using a sterile 200 μL pipette tip. Detached cells and cellular debris were removed by washing the wells twice with phosphate-buffered saline (PBS). The cells were then maintained in serum-free DMEM and assigned to the following experimental groups: untreated control, EGCG (100 μM), DOX (1.5 μM), and EGCG (100 μM) + DOX (1.5 μM). The treatment concentrations corresponded to the working concentrations defined in Section 2.2.

Images of the wound area were acquired immediately after scratching (0 h) and after 24 h using an inverted microscope under identical magnification and imaging conditions. Wound areas were quantified using ImageJ software (version 1.54; National Institutes of Health, Bethesda, MD, USA). Wound closure was calculated relative to the initial wound area at 0 h using the following equation:

Wound closure (%) = [(A_0_ − A_24_)/A_0_] × 100,

where A_0_ represents the wound area immediately after scratching, and A_24_ represents the wound area after 24 h.

The assay was conducted in three independent biological experiments (*n* = 3), with one well allocated to each treatment group per experiment. Three non-overlapping microscopic fields were evaluated from each well, and the mean of the three fields was considered the experimental value for that biological replicate. Accordingly, statistical analyses were performed using the three independent biological replicate values rather than individual microscopic fields.

### 2.6. Flow-Cytometric Profiling of Treatment-Induced Apoptotic Populations

Apoptotic cell populations were quantified by Annexin V-FITC/propidium iodide (PI) double staining using an Annexin V-FITC Apoptosis Detection Kit (BD Biosciences, San Jose, CA, USA). HeLa cells were seeded into 6-well plates at a density of 2 × 10^5^ cells/well and allowed to adhere before treatment. Cells were assigned to untreated control, EGCG (100 μM), DOX (1.5 μM), and EGCG (100 μM) + DOX (1.5 μM) groups and incubated for 48 h. The treatment concentrations corresponded to the working concentrations defined in Section 2.2.

Following treatment, cells were harvested by trypsinization using 0.25% trypsin–EDTA, washed twice with cold PBS (PBS; pH 7.4), and resuspended in 1× binding buffer at a density of 1 × 10^6^ cells/mL. An aliquot of 100 μL of the cell suspension, corresponding to approximately 1 × 10^5^ cells, was transferred to each staining tube and incubated with 5 μL of Annexin V-FITC and 5 μL of PI for 15 min at room temperature in the dark. Subsequently, 400 μL of 1× binding buffer was added to each tube, and the samples were analyzed within 1 h using a FACSCalibur flow cytometer (BD Biosciences).

For each biological replicate, a minimum of 20,000 events per sample were acquired, and flow cytometric data were analyzed using FlowJo software (version 10.8.1; BD Biosciences, Franklin Lakes, NJ, USA). Cell populations were classified according to Annexin V-FITC and PI staining as viable (Annexin V^−^/PI^−^), early apoptotic (Annexin V^+^/PI^−^), late apoptotic (Annexin V^+^/PI^+^), or necrotic (Annexin V^−^/PI^+^). Total apoptotic cells were defined as the sum of the early- and late-apoptotic populations.

### 2.7. Image-Based Cytometric Evaluation of Treatment-Associated Cell Death

Treatment-associated changes in cell viability and cell death were further evaluated using the Tali™ Image-Based Cytometer (Thermo Fisher Scientific, Waltham, MA, USA). HeLa cells were seeded into 6-well plates and assigned to untreated control, EGCG (100 μM), DOX (1.5 μM), and EGCG (100 μM) + DOX (1.5 μM) groups. Following 48 h of treatment, both adherent and floating cells were collected to ensure inclusion of treatment-detached cells. The collected cells were washed twice with PBS and prepared for image-based cytometric analysis.

Cell populations were evaluated using fluorescence-based image cytometry, and representative images were acquired for each experimental group. Viable and PI-positive dead-cell populations were quantified and expressed as percentages of the analyzed cell population. The analysis was performed using identical acquisition and analysis settings across all experimental groups.

The Sub-G1 fraction presented together with the image-based cytometric findings was derived from the PI-based DNA-content analysis described separately in the cell-cycle analysis section and was not considered an Annexin V-defined apoptotic population.

### 2.8. Image-Based DNA Content Profiling and Cell-Cycle Distribution

Cell-cycle distribution was evaluated by PI-based DNA-content analysis using a Tali™ Image-Based Cytometer (Invitrogen, Carlsbad, CA, USA). HeLa cells were assigned to untreated control, EGCG (100 μM), DOX (1.5 μM), and EGCG (100 μM) + DOX (1.5 μM) treatment groups. Following 48 h of treatment, cells were collected and washed twice with cold PBS. The resulting cell pellets were gently resuspended in ice-cold 70% ethanol and fixed overnight at 4 °C.

Following fixation, cells were washed to remove residual ethanol and resuspended at approximately 1 × 10^6^ cells/mL in PI/RNase staining solution containing 50 μg/mL PI (Sigma-Aldrich, St. Louis, MO, USA) and 100 μg/mL RNase A (Sigma-Aldrich, St. Louis, MO, USA). Samples were incubated for 30 min at room temperature in the dark before image-based cytometric analysis. A minimum of 5000 cells were analyzed per sample using identical acquisition and analysis settings across all experimental groups.

Cell populations were classified according to DNA content into G0/G1, S, and G2/M phases. The hypodiploid Sub-G1 population was additionally quantified as an apoptosis-associated DNA-content fraction. The experiment was performed in three independent biological replicates (*n* = 3), and cell-cycle distributions are reported as the percentage of cells within each phase.

### 2.9. DCFH-DA-Based Fluorescence Imaging and Quantitative Assessment of Intracellular Oxidative Stress and NAC Modulation

Intracellular oxidative stress was evaluated using 2′,7′-dichlorofluorescein diacetate (DCFH-DA; Sigma-Aldrich, St. Louis, MO, USA). HeLa cells were assigned to eight experimental groups: untreated control, NAC (5 mM), EGCG (100 μM), DOX (1.5 μM), EGCG (100 μM) + DOX (1.5 μM), NAC + EGCG, NAC + DOX, and NAC + EGCG + DOX. For NAC-containing groups, cells were pretreated with 5 mM NAC for 2 h before exposure to EGCG, DOX, or their combination. Cells were subsequently incubated with the indicated treatments for 48 h.

For fluorescence imaging, HeLa cells were seeded onto sterile glass coverslips placed in 24-well plates and allowed to adhere for 24 h. Following treatment, cells were gently washed twice with PBS and incubated with 10 μM DCFH-DA prepared in serum-free DMEM for 30 min at 37 °C in the dark. Excess probe was removed by washing twice with PBS, and intracellular DCF-associated green fluorescence was immediately visualized using an inverted fluorescence microscope. Representative images were acquired using identical magnification, exposure time, illumination intensity, camera gain, and image-processing parameters for all experimental groups to allow direct qualitative comparison of intracellular ROS-associated fluorescence.

For quantitative analysis, parallel experiments were performed in black 96-well plates, with HeLa cells seeded at a density of 2 × 10^4^ cells/well. Following the same treatment and DCFH-DA staining procedures, fluorescence intensity was measured using a fluorescence microplate reader at excitation and emission wavelengths of 485 and 530 nm, respectively. To account for treatment-associated differences in viable cell number, DCFH-DA fluorescence values were normalized to the corresponding cell viability values obtained from parallel CCK-8 assays performed under identical experimental conditions. Normalized fluorescence values were expressed as fold change relative to the untreated control group, which was set to 1.0.

All fluorescence measurements were obtained from three independent biological experiments (*n* = 3). The three individual data points displayed for each experimental group in the quantitative analysis represent the values obtained from these three independent biological replicates, and data are presented as mean ± SD. An independent positive-control condition for DCFH-DA oxidation, such as exogenous H_2_O_2_ exposure, was not included in the present experimental design. Statistical comparisons were performed as described in the statistical analysis section.

### 2.10. Immunocytochemical Characterization and Quantification of Caspase-3 Immunoreactivity

HeLa cells were seeded onto sterile glass coverslips and assigned to untreated control, EGCG (100 μM), DOX (1.5 μM), and EGCG (100 μM) + DOX (1.5 μM) groups. Following 48 h of treatment, cells were fixed with 4% paraformaldehyde for 15 min, permeabilized with 0.1% Triton X-100 for 10 min, and blocked with 5% bovine serum albumin (BSA) for 1 h at room temperature.

The cells were incubated overnight at 4 °C with a rabbit monoclonal anti-Caspase-3 primary antibody (Abcam, Cambridge, UK; Cat. No. ab184787), which recognizes total Caspase-3. Following washing with PBS, cells were incubated with an HRP-conjugated secondary antibody for 1 h at room temperature. Immunoreactivity was visualized using 3,3′-diaminobenzidine (DAB) as the chromogenic substrate, and nuclei were counterstained with Mayer’s hematoxylin.

Bright-field images were acquired under identical microscope, magnification, illumination, and camera settings for all experimental groups. Caspase-3 immunoreactivity was quantified using ImageJ software (National Institutes of Health, Bethesda, MD, USA). Following background subtraction, the integrated optical density of DAB-positive staining was measured and expressed in arbitrary units (a.u.). At least five randomly selected, non-overlapping microscopic fields were analyzed per well. The mean value obtained from these fields was considered the experimental value for the corresponding biological replicate. The experiment was independently performed three times (*n* = 3), and the three independent biological replicate values were used for statistical analysis. Data are presented as mean ± SD.

### 2.11. Transcriptional Profiling of Apoptosis- and Survival-Related Genes by RT-qPCR

HeLa cells were assigned to untreated control, EGCG (100 μM), DOX (1.5 μM), and EGCG (100 μM) + DOX (1.5 μM) groups and treated for 48 h. Total RNA was isolated using TRIzol reagent (Invitrogen, Carlsbad, CA, USA) according to the manufacturer’s instructions. RNA concentration and purity were assessed using a NanoDrop 2000 spectrophotometer (Thermo Fisher Scientific, Waltham, MA, USA), and RNA purity was evaluated based on the A260/A280 absorbance ratio. Complementary DNA (cDNA) was synthesized from the isolated RNA using the High-Capacity cDNA Reverse Transcription Kit (Applied Biosystems, Foster City, CA, USA) according to the manufacturer’s protocol.

Quantitative real-time PCR was performed using SYBR Green Master Mix (Bio-Rad, Hercules, CA, USA) on a StepOnePlus Real-Time PCR System (Applied Biosystems, Foster City, CA, USA). Gene-specific primers targeting *EGFR*, *FOXP3*, *CASP3*, *CASP7*, and the reference gene *GAPDH* were designed using NCBI Primer-BLAST (https://www.ncbi.nlm.nih.gov/tools/primer-blast/, accessed on 1 August 2026). The forward and reverse primer sequences used for each target gene are provided in Table 1.

The amplification protocol consisted of an initial denaturation step at 95 °C for 10 min, followed by 40 amplification cycles comprising denaturation at 95 °C for 15 s and annealing/extension at 60 °C for 60 s. Primer specificity was verified by melting-curve analysis following amplification, with a single distinct melting peak considered indicative of specific amplification. Primer amplification efficiencies were evaluated using standard curves generated from serially diluted cDNA, and primer sets exhibiting amplification efficiencies within the acceptable range of 90–110% were used for relative gene-expression analysis. Relative gene-expression levels were normalized to *GAPDH* and calculated using the 2^−ΔΔCt^ method. The untreated control group was used as the calibrator and assigned a relative expression value of 1; accordingly, transcript levels in the treatment groups were expressed as fold changes relative to the untreated control.

The experiments were performed in three independent biological replicates (*n* = 3), and the resulting relative expression values were used for statistical analysis.

### 2.12. Network-Based Functional Contextualization of Treatment-Responsive Genes

Bioinformatic analyses were performed to provide a predictive functional context for the RT-qPCR findings rather than to serve as experimental validation of molecular mechanisms. The treatment-responsive genes evaluated by RT-qPCR (*EGFR*, *FOXP3*, *CASP3*, and *CASP7*) were used as the input gene set for subsequent network and functional enrichment analyses.

Protein–protein interaction (PPI) analysis was performed using the STRING database (version 12.0; https://string-db.org/, accessed on 1 August 2026), with Homo sapiens selected as the reference organism. The minimum required interaction score was set to 0.700 (high confidence). Active interaction sources included experimentally determined interactions, curated databases, co-expression, text mining, gene neighborhood, gene fusion, and gene co-occurrence. Disconnected nodes were excluded from the final network visualization. The resulting interaction network was exported to Cytoscape version 3.10.2 for visualization and network analysis. Potential hub genes were identified using the cytoHubba plugin based on Degree centrality, with nodes displaying the highest Degree values considered the most highly connected components of the network. Lower-connectivity proteins were retained as secondary interacting nodes to preserve the broader network context.

Functional enrichment analysis was performed using the STRING functional enrichment module. Gene Ontology (GO) enrichment was evaluated across the Biological Process (BP), Cellular Component (CC), and Molecular Function (MF) categories, together with Kyoto Encyclopedia of Genes and Genomes (KEGG) pathway enrichment. Enrichment significance was evaluated using the Benjamini–Hochberg false discovery rate (FDR) correction, and terms with an FDR-adjusted *p* value < 0.05 were considered significantly enriched.

Because the network and enrichment analyses were derived from a limited set of treatment-responsive genes and database-predicted functional associations, these analyses were interpreted as exploratory and hypothesis-generating. Accordingly, PPI connectivity, hub-gene ranking, and GO/KEGG enrichment were used to contextualize the experimental RT-qPCR findings and to identify potentially relevant biological associations for future investigation rather than as evidence of experimentally validated molecular mechanisms.

### 2.13. Statistical Framework and Quantitative Data Analysis

All experiments were performed in three independent biological replicates (*n* = 3). Where applicable, technical replicate measurements were obtained within each biological experiment and averaged to generate a single value for the corresponding biological replicate. Similarly, when multiple microscopic fields were evaluated within the same experimental sample, field-level measurements were averaged before statistical analysis and were not treated as independent biological replicates. Data are presented as mean ± standard deviation (SD).

Statistical analyses were performed using GraphPad Prism version 8.0 (GraphPad Software, San Diego, CA, USA). For the Calcein-AM/PI viability and membrane-integrity analysis, wound-closure assay, Annexin V-FITC/PI apoptosis analysis, image-based cytometric cell-death assessment, cell-cycle analysis, DCFH-DA fluorescence analysis, caspase-3 immunocytochemical quantification, and RT-qPCR analysis, comparisons among the relevant experimental groups were performed using one-way analysis of variance (ANOVA), followed by Tukey’s multiple-comparisons post hoc test. Tukey’s procedure was used to control for multiple pairwise comparisons within each individual experimental analysis. No additional global correction for multiple testing across distinct biological endpoints or separate experiments was applied, because these endpoints were analyzed as predefined, biologically distinct experimental outcomes rather than as a single family of simultaneous hypothesis tests. A two-sided *p* value < 0.05 was considered statistically significant.

Concentration–response curves were analyzed by nonlinear regression using a log(inhibitor) versus normalized response model with a variable slope, and IC_50_ values were estimated from the fitted curves. Drug-interaction analyses were performed according to the Chou–Talalay method using CompuSyn software (version 3.0.1; ComboSyn Inc., Paramus, NJ, USA), as described in Section 2.3. CI values were interpreted using the predefined thresholds of CI < 0.90 for synergism, 0.90 ≤ CI ≤ 1.10 for an additive interaction, and CI > 1.10 for antagonism.

## 3. Results

### 3.1. Concentration- and Time-Dependent Cytotoxicity of EGCG and DOX

**Figure 1:**
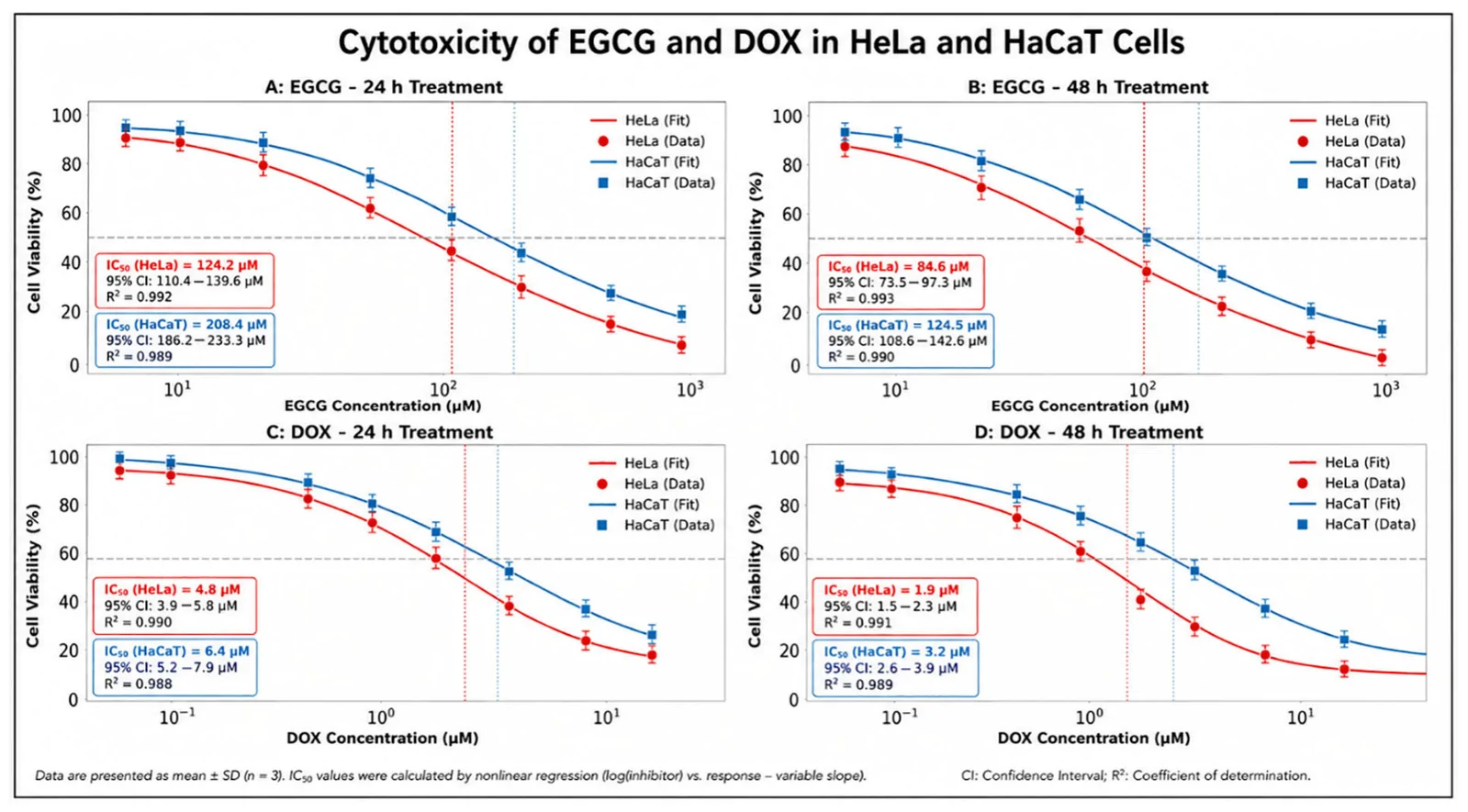
Concentration–response effects of EGCG and DOX on HeLa and HaCaT cell viability after 24 and 48 h of treatment. Concentration–response curves show the effects of EGCG (**A**,**B**) and DOX (**C**,**D**) on HeLa and HaCaT cells following 24 h (**A**,**C**) and 48 h (**B**,**D**) exposure. Cell viability was determined using the CCK-8 assay and expressed relative to the untreated control. Data are presented as mean ± SD from three independent biological experiments (*n* = 3). IC_50_ values were estimated by nonlinear regression using a log(inhibitor) versus normalized response model with a variable slope and are presented with 95% confidence intervals (CI) and coefficients of determination (R^2^). Horizontal dashed lines indicate 50% cell viability, and vertical dotted lines indicate the calculated IC_50_ values for HeLa and HaCaT cells.

The cytotoxic effects of EGCG and DOX on HeLa and HaCaT cells were evaluated after 24 and 48 h of exposure using the CCK-8 assay. Both compounds produced concentration- and time-dependent reductions in cell viability, with HeLa cells exhibiting slightly higher sensitivity than HaCaT cells (Figure 1).

In HeLa cells, the IC_50_ value for EGCG decreased from 124.2 μM (95% CI: 110.4–139.6 μM) at 24 h to 84.6 μM (95% CI: 73.5–97.3 μM) at 48 h. A similar time-dependent shift was observed for DOX, with IC_50_ values decreasing from 4.8 μM (95% CI: 3.9–5.8 μM) at 24 h to 1.9 μM (95% CI: 1.5–2.3 μM) at 48 h.

HaCaT cells also showed concentration- and time-dependent reductions in viability following exposure to both compounds but exhibited consistently higher IC_50_ values than HeLa cells. For EGCG, the IC_50_ values were 208.4 μM (95% CI: 186.2–233.3 μM) at 24 h and 124.5 μM (95% CI: 108.6–142.6 μM) at 48 h. For DOX, the corresponding IC_50_ values were 6.4 μM (95% CI: 5.2–7.9 μM) and 3.2 μM (95% CI: 2.6–3.9 μM), respectively. Collectively, these concentration–response profiles indicate a slight differential sensitivity between the two cell lines, with lower IC_50_ values observed in HeLa cells than in the HaCaT comparator cell line.

### 3.2. Combined Effects of EGCG and DOX on Cell Viability and Drug Interaction

Cell viability following combined EGCG and DOX exposure was evaluated in both HeLa and HaCaT cells across increasing combination concentrations. Under the 1:2 relative-dose scheme, increasing EGCG + DOX concentrations produced a progressive reduction in cell viability in both cell lines (Table 2). In HeLa cells, viability decreased from 43.0% at 12.5 μM EGCG + 0.25 μM DOX to 8.0% at 200 μM EGCG + 4.00 μM DOX. Over the same concentration range, HaCaT cell viability decreased from 87.0% to 52.0% (Table 2).

Drug-interaction analysis further demonstrated distinct interaction profiles between the two cell lines. In HeLa cells, CI values of 0.75 (Fa = 0.68), 0.62 (Fa = 0.78), and 0.88 (Fa = 0.60) were obtained for the 1:1, 1:2, and 2:1 relative-dose combination schemes, respectively, indicating synergistic interactions under all three tested conditions. In HaCaT cells, the corresponding CI values were 1.15 (Fa = 0.35), 1.32 (Fa = 0.30), and 0.95 (Fa = 0.40), indicating antagonistic interactions under the 1:1 and 1:2 relative-dose combination schemes and an additive interaction under the 2:1 relative-dose combination scheme. The analysis was based on three independent biological experiments (*n* = 3). The reported CI values therefore represent interaction estimates at the corresponding experimentally observed Fa levels rather than at a single prespecified Fa level.

### 3.3. Differential Sensitivity of HeLa and HaCaT Cells

**Figure 2:**
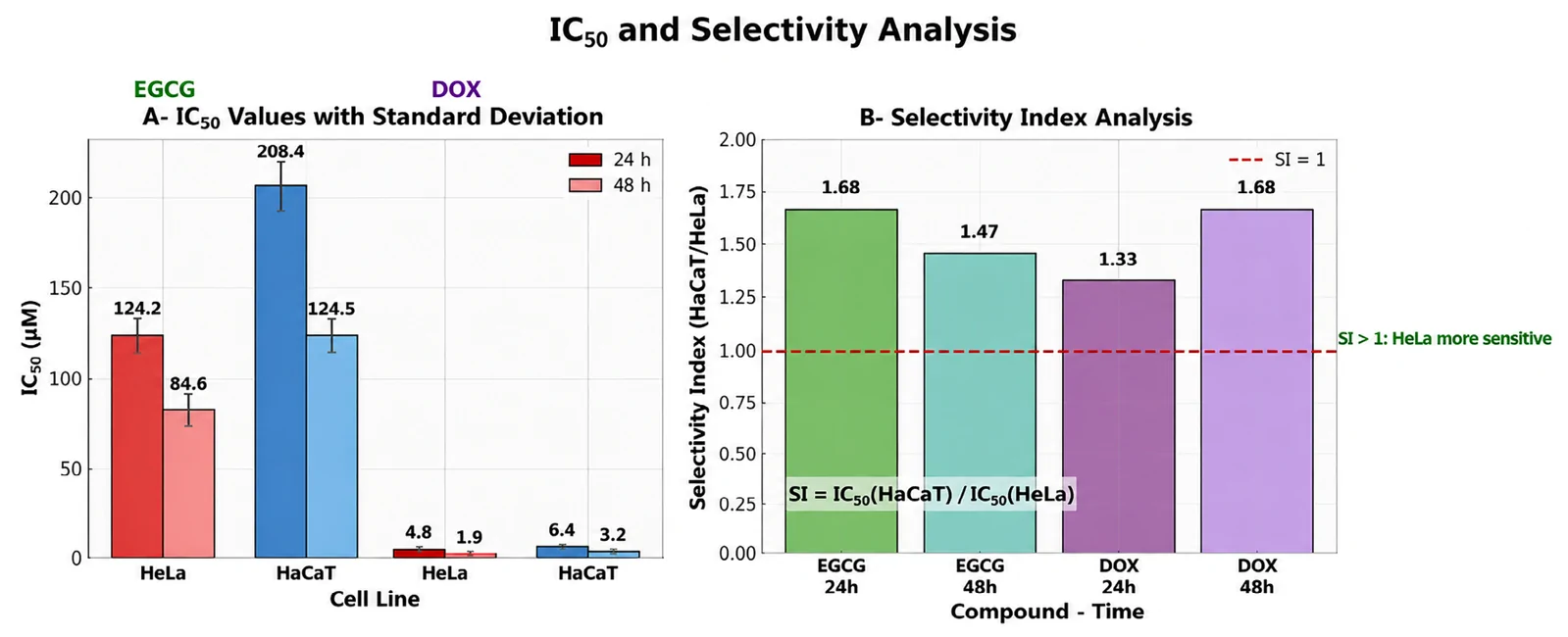
IC_50_ values and differential sensitivity of HeLa and HaCaT cells to EGCG and DOX. (**A**) Comparison of the half-maximal inhibitory concentration (IC_50_) values of EGCG and DOX in HeLa and HaCaT cells following 24 and 48 h of treatment. IC_50_ values were estimated by nonlinear regression analysis of CCK-8 cell viability data and are presented as mean ± SD from three independent biological experiments (*n* = 3). (**B**) SI calculated as IC_50_(HaCaT)/IC_50_(HeLa) for each compound and treatment duration. The dashed horizontal line indicates SI = 1, with values > 1 indicating greater sensitivity of HeLa cells relative to HaCaT cells. The calculated SI values ranged from 1.33 to 1.68, indicating modest differential sensitivity of HeLa cells to EGCG and DOX rather than cancer-specific selectivity.

The selectivity index (SI), calculated as IC_50_(HaCaT)/IC_50_(HeLa), was used to further characterize differences in sensitivity between HeLa and HaCaT cells. For EGCG, the SI values were 1.68 and 1.47 at 24 and 48 h, respectively, whereas the corresponding values for DOX were 1.33 and 1.68. All calculated SI values were greater than 1, consistent with greater sensitivity of HeLa cells to both compounds relative to the non-malignant HaCaT comparator cells. However, the relatively modest SI values (1.33–1.68) indicate modest differential sensitivity rather than cancer-specific selectivity (Figure 2).

### 3.4. Differential EGCG–DOX Drug Interactions in HeLa and HaCaT Cells

**Figure 3:**
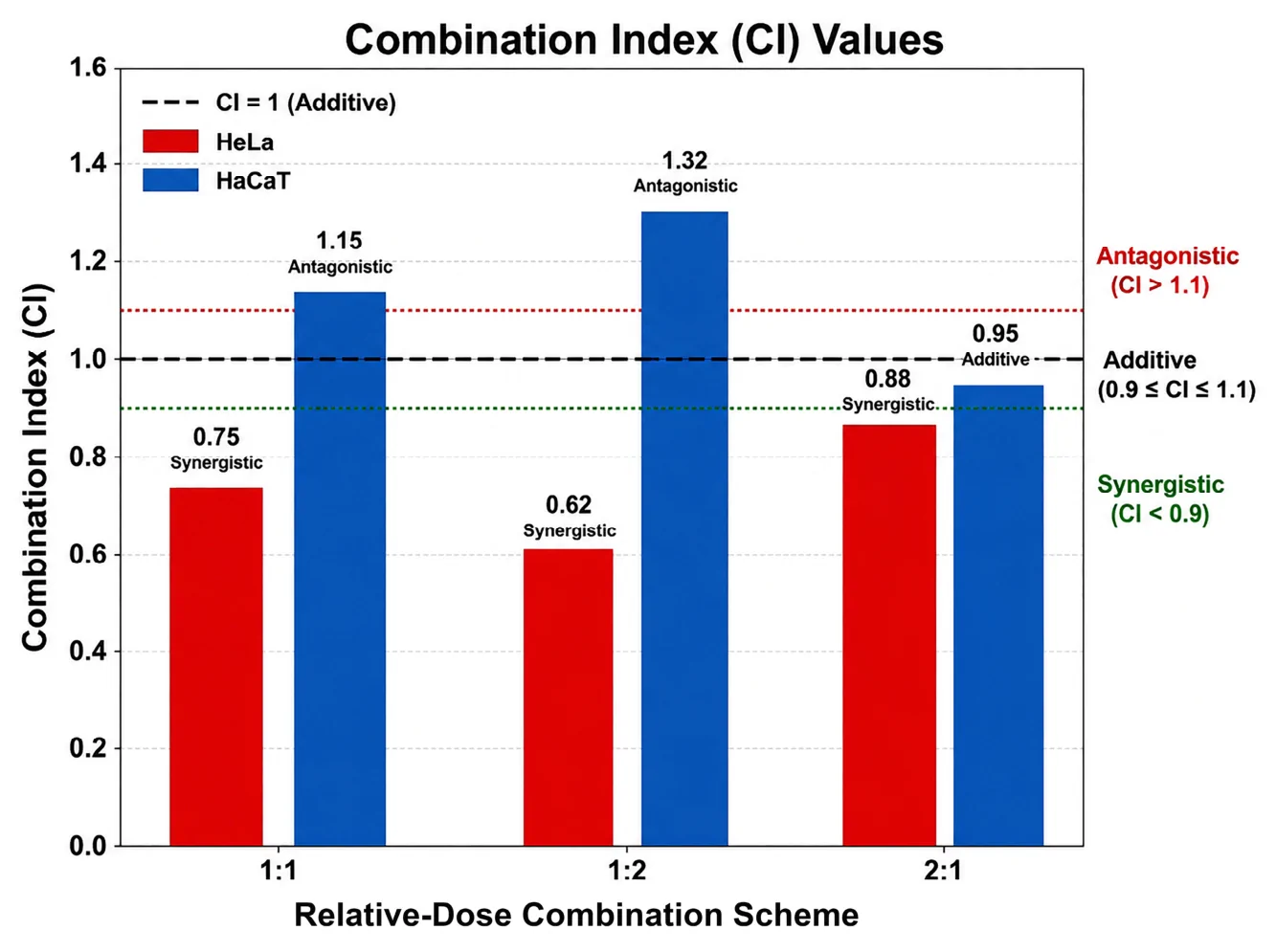
CI analysis of EGCG and DOX interactions in HeLa and HaCaT cells. CI values were determined using the Chou–Talalay method for three predefined relative-dose combination schemes designated 1:1, 1:2, and 2:1. These designations represent relative dose proportions within the experimental combination design and do not correspond to direct molar ratios between EGCG and DOX. In HeLa cells, CI values of 0.75 (Fa = 0.68), 0.62 (Fa = 0.78), and 0.88 (Fa = 0.60) were obtained for the 1:1, 1:2, and 2:1 relative-dose combination schemes, respectively, indicating synergistic interactions under all three tested conditions. In HaCaT cells, the corresponding CI values were 1.15 (Fa = 0.35), 1.32 (Fa = 0.30), and 0.95 (Fa = 0.40), indicating antagonistic interactions under the 1:1 and 1:2 relative-dose combination schemes and an additive interaction under the 2:1 relative-dose combination scheme. The analysis was based on three independent biological experiments (*n* = 3). The reported CI values therefore represent interaction estimates at the corresponding experimentally observed Fa levels rather than at a single prespecified Fa level.

Drug interactions between EGCG and DOX were evaluated using the Chou–Talalay CI method across three predefined relative-dose combination schemes, designated 1:1, 1:2, and 2:1. In HeLa cells, all three combination schemes yielded CI values below 0.90, indicating synergistic interactions. The lowest CI value was observed for the 1:2 scheme (CI = 0.62), followed by the 1:1 (CI = 0.75) and 2:1 (CI = 0.88) schemes (Figure 3).

In contrast, the interaction profile differed in HaCaT cells. The 1:1 and 1:2 schemes yielded CI values of 1.15 and 1.32, respectively, indicating antagonistic interactions, whereas the 2:1 scheme yielded a CI value of 0.95, corresponding to an additive interaction. Thus, under the tested combination conditions, synergistic EGCG–DOX interactions were observed in HeLa cells, whereas comparable synergism was not observed in HaCaT cells (Figure 3).

### 3.5. Orthogonal Assessment of Cell Viability and Membrane Integrity by Calcein-AM/PI Staining

**Figure 4:**
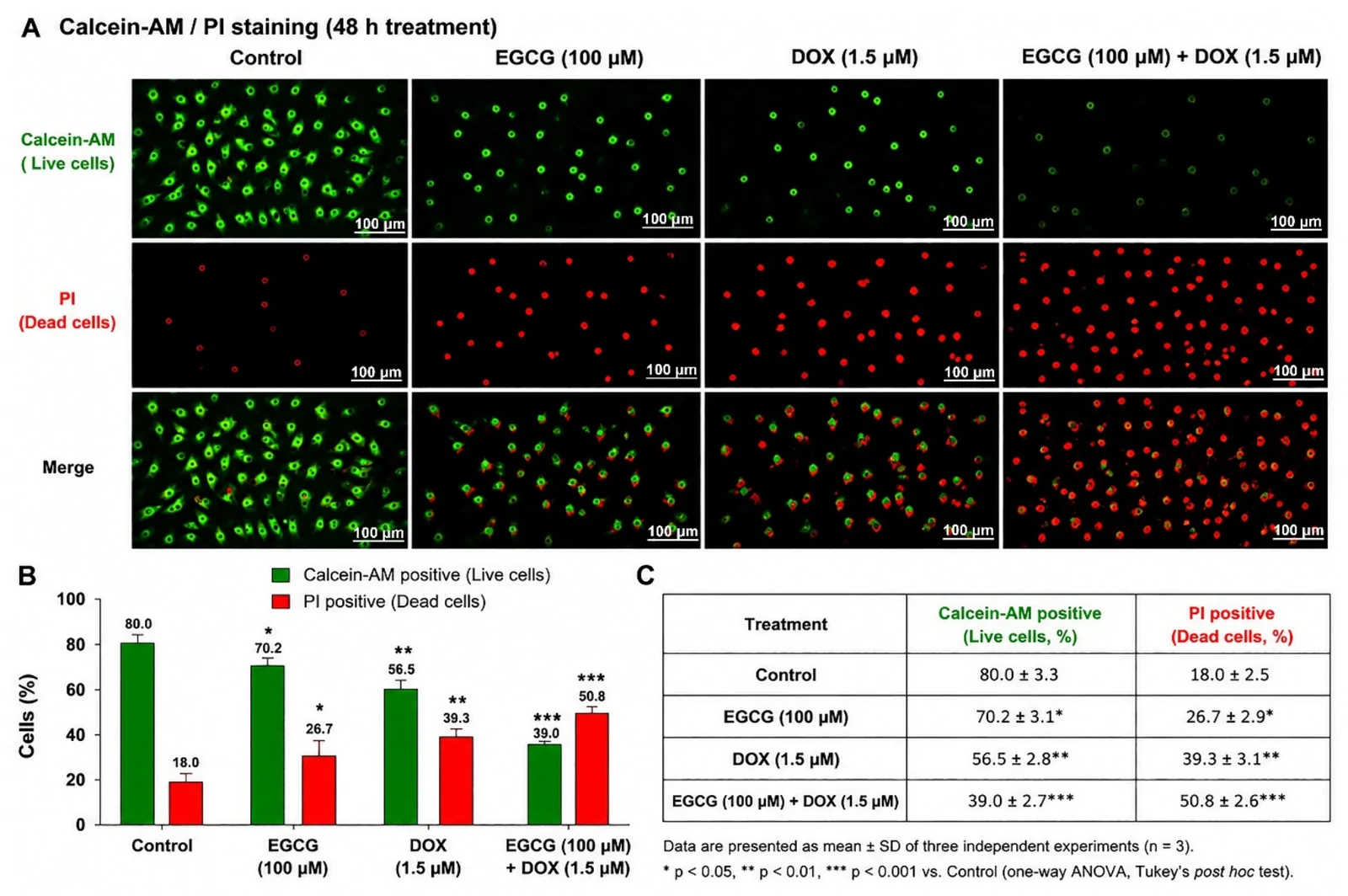
Fluorescence-based assessment of cell viability and membrane integrity by Calcein-AM/PI staining in HeLa cells following EGCG and DOX treatment. (**A**) Representative fluorescence images of HeLa cells after 48 h of treatment with EGCG (100 μM), DOX (1.5 μM), or their combination (100 μM EGCG + 1.5 μM DOX), compared with the untreated control. Calcein-AM-associated green fluorescence indicates viable cells with preserved intracellular esterase activity and membrane integrity, whereas PI-associated red fluorescence indicates cells with compromised plasma membrane integrity. Merged images show the relative distribution of Calcein-AM- and PI-positive cells within the same microscopic fields. Scale bars = 100 μm. (**B**) Quantitative analysis of Calcein-AM-positive viable cells and PI-positive membrane-compromised cells. (**C**) Summary of the corresponding quantitative values. The proportion of Calcein-AM-positive cells decreased from 80.0 ± 3.3% in the control group to 70.2 ± 3.1%, 56.5 ± 2.8%, and 39.0 ± 2.7% following EGCG, DOX, and combined EGCG + DOX treatment, respectively. Conversely, PI-positive cells increased from 18.0 ± 2.5% in the control group to 26.7 ± 2.9%, 39.3 ± 3.1%, and 50.8 ± 2.6%, respectively. Data are presented as mean ± SD from three independent biological experiments (*n* = 3). Statistical significance was evaluated using one-way ANOVA followed by Tukey’s multiple-comparisons post hoc test. * *p* < 0.05, ** *p* < 0.01, and *** *p* < 0.001 versus the untreated control.

To independently evaluate treatment-associated changes in cell viability and membrane integrity using an assay distinct from the CCK-8 metabolic readout, HeLa cells were subjected to Calcein-AM/PI dual-fluorescence staining after 48 h of treatment with EGCG, DOX, or their combination (Figure 4). Representative fluorescence images demonstrated a predominantly Calcein-AM-positive viable-cell population in the untreated control group, with relatively few PI-positive cells. EGCG treatment resulted in a moderate reduction in Calcein-AM-positive cells accompanied by an increase in PI-positive cells, whereas DOX treatment produced a more pronounced shift toward reduced viable-cell staining and increased PI positivity. The greatest alteration was observed following combined EGCG + DOX treatment, which exhibited the lowest Calcein-AM-positive cell proportion and the highest PI-positive cell proportion among the experimental groups (Figure 4A).

Quantitative analysis was consistent with the representative fluorescence images (Figure 4B,C). The proportion of Calcein-AM-positive viable cells was 80.0 ± 3.3% in the untreated control group and decreased to 70.2 ± 3.1% following EGCG treatment, 56.5 ± 2.8% following DOX treatment, and 39.0 ± 2.7% following combined EGCG + DOX treatment. Conversely, the proportion of PI-positive membrane-compromised/dead cells increased from 18.0 ± 2.5% in the control group to 26.7 ± 2.9%, 39.3 ± 3.1%, and 50.8 ± 2.6% following EGCG, DOX, and combined treatment, respectively. Thus, the combination group showed the greatest reduction in Calcein-AM-positive viable cells together with the greatest increase in PI-positive membrane-compromised cells.

These fluorescence-based findings were directionally consistent with the treatment-associated reduction in cell viability observed using the CCK-8 assay. Because Calcein-AM/PI staining evaluates intracellular esterase activity and plasma membrane integrity rather than the metabolic reduction measured by CCK-8, the results provide complementary orthogonal evidence supporting the treatment-associated loss of viability observed following EGCG and DOX exposure. The most pronounced changes were observed following combined treatment.

### 3.6. Treatment-Associated Reduction in Wound Closure in HeLa Cells

**Figure 5:**
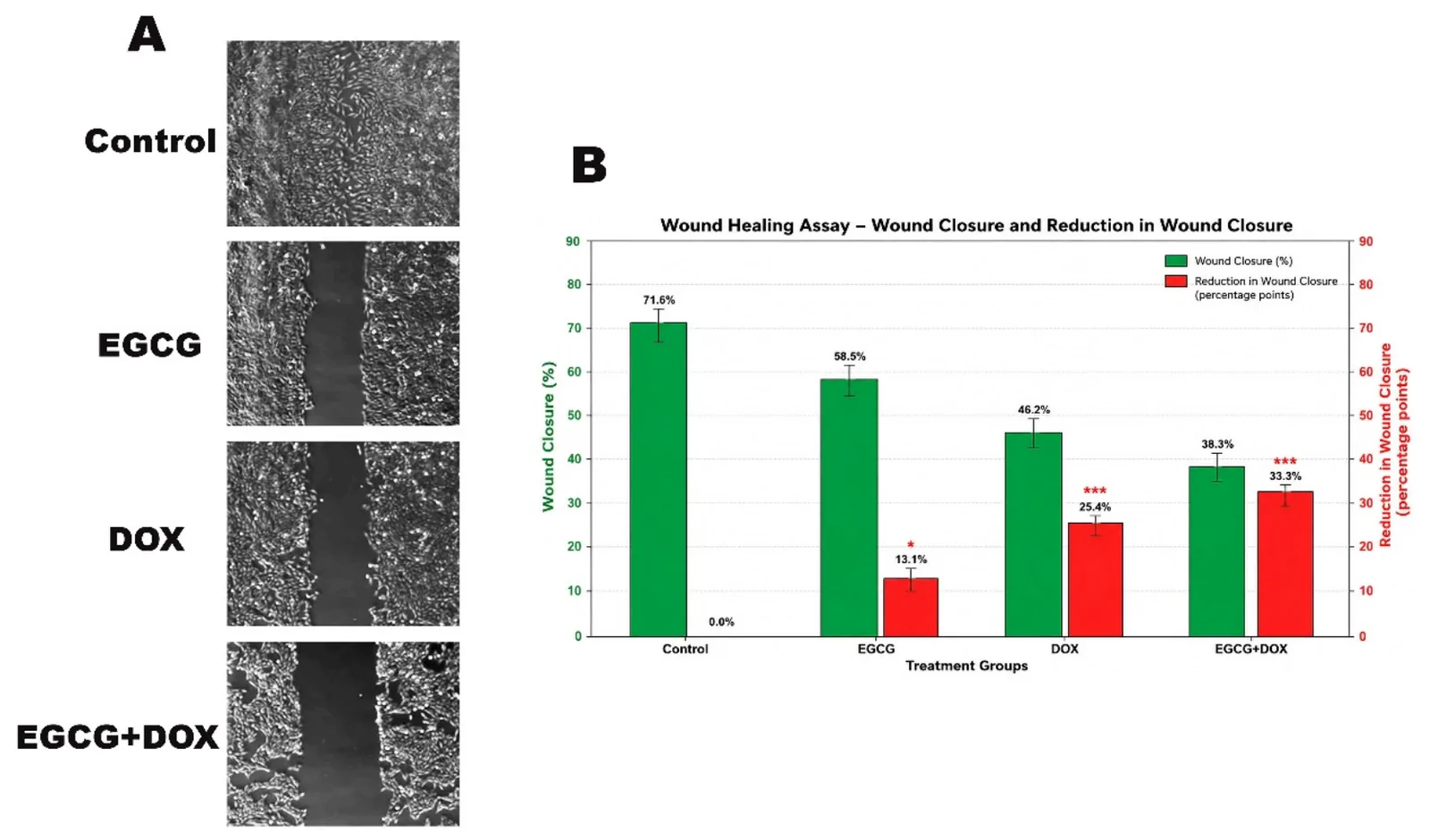
Effect of EGCG and DOX treatment on wound closure in HeLa cells. (**A**) Representative wound-healing images of HeLa cells after 24 h of treatment with EGCG (100 μM), DOX (1.5 μM), or the EGCG + DOX combination (100 μM + 1.5 μM), compared with the untreated control. (**B**) Quantitative analysis of wound closure and the corresponding absolute reduction in wound closure relative to the untreated control. Wound closure was 71.6 ± 5.2%, 58.5 ± 4.1%, 46.2 ± 3.6%, and 38.3 ± 3.7% in the control, EGCG, DOX, and EGCG + DOX groups, respectively. Relative to the untreated control, EGCG, DOX, and EGCG + DOX reduced wound closure by 13.1, 25.4, and 33.3 percentage points, respectively. Data are presented as mean ± SD from three independent biological experiments (*n* = 3). Statistical significance was evaluated using one-way ANOVA followed by Tukey’s multiple-comparisons test. * *p* < 0.05 and *** *p* < 0.001 versus the untreated control.

The migratory behavior of HeLa cells was evaluated using a wound-healing assay over a 24 h period. Untreated control cells exhibited 71.6 ± 5.2% wound closure. EGCG treatment reduced wound closure to 58.5 ± 4.1%, whereas DOX treatment resulted in a further reduction to 46.2 ± 3.6%. The lowest wound closure was observed following combined EGCG + DOX treatment, reaching 38.3 ± 3.7% (Figure 5).

Relative to the untreated control, these values corresponded to absolute reductions in wound closure of 13.1, 25.4, and 33.3 percentage points for EGCG, DOX, and EGCG + DOX, respectively. The 33.3-percentage-point difference observed with the combination corresponded to an approximately 46.5% relative reduction in wound closure compared with the control. Collectively, these findings indicate that EGCG and DOX reduced wound closure under the experimental conditions, with the greatest reduction observed following combined treatment.

### 3.7. EGCG and DOX Increase Apoptotic Cell Populations in HeLa Cells

**Figure 6:**
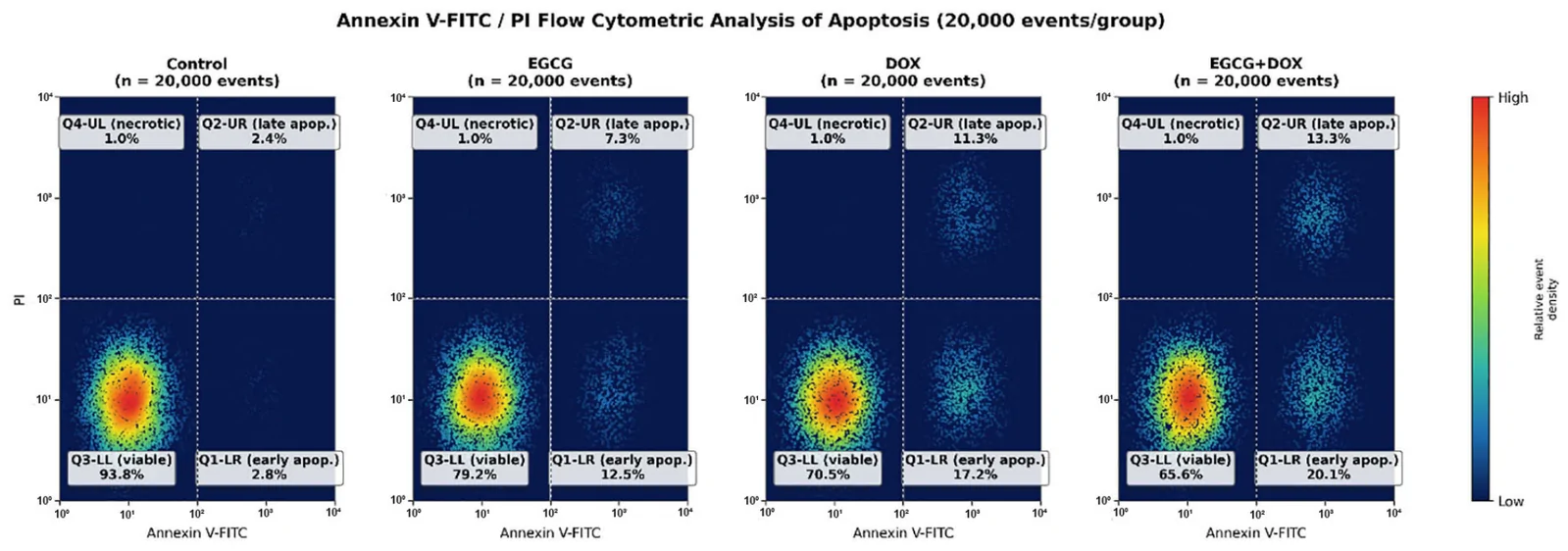
Representative Annexin V-FITC/PI flow cytometry plots showing the distribution of viable, early apoptotic, late apoptotic, and necrotic HeLa cells after 48 h of treatment with EGCG (100 μM), DOX (1.5 μM), or their combination. Cells were stained with Annexin V-FITC and PI and analyzed by flow cytometry, with 20,000 events recorded per sample. Quadrant populations were defined as follows: Q3-LL (Annexin V^−^/PI^−^), viable cells; Q1-LR (Annexin V^+^/PI^−^), early apoptotic cells; Q2-UR (Annexin V^+^/PI^+^), late apoptotic cells; and Q4-UL (Annexin V^−^/PI^+^), necrotic cells. Representative plots show increases in the early and late apoptotic populations following EGCG and DOX treatment, accompanied by decreases in the viable cell population. The EGCG + DOX group exhibited the highest proportions of early (20.1%) and late (13.3%) apoptotic cells and the lowest proportion of viable cells (65.6%). Representative plots from three independent biological experiments (*n* = 3) are shown.

The apoptotic response of HeLa cells to EGCG (100 μM), DOX (1.5 μM), and their combination was evaluated after 48 h using Annexin V-FITC/PI flow cytometry. Representative quadrant analysis showed that the total apoptotic population, defined as the sum of early apoptotic (Annexin V^+^/PI^−^) and late apoptotic (Annexin V^+^/PI^+^) cells, increased from 5.2% in the untreated control to 19.8% following EGCG treatment and 28.5% following DOX treatment. The highest total apoptotic fraction was observed in the EGCG + DOX group, reaching 33.4%, corresponding to an approximately 6.4-fold increase relative to the control (Figure 6).

Analysis of the individual apoptotic populations showed that early apoptosis increased from 2.8% in the control group to 12.5%, 17.2%, and 20.1% following EGCG, DOX, and EGCG + DOX treatment, respectively. Similarly, the late apoptotic population increased from 2.4% in the control to 7.3%, 11.3%, and 13.3%, respectively. Concomitantly, the viable cell population decreased from 93.8% in the control group to 79.2%, 70.5%, and 65.6% following EGCG, DOX, and combined treatment, respectively. These findings demonstrate a progressive increase in apoptotic cell populations, with the highest apoptotic fraction observed following combined EGCG + DOX treatment.

### 3.8. EGCG and DOX Alter Cell-Cycle Distribution in HeLa Cells

**Figure 7:**
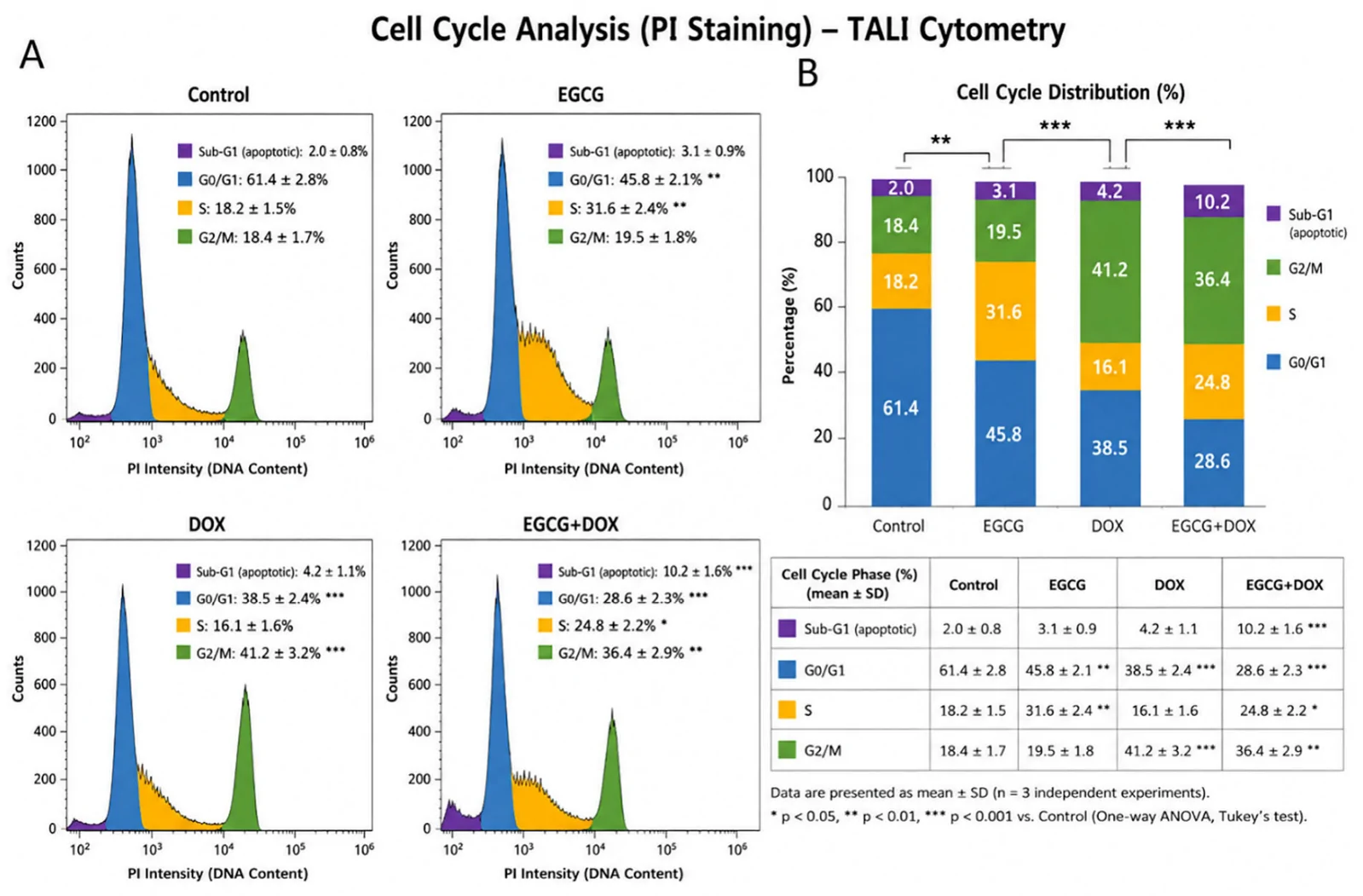
Effects of EGCG and DOX on cell-cycle distribution in HeLa cells. (**A**) Representative PI-stained DNA-content histograms obtained by TALI image-based cytometry after 48 h of treatment with EGCG (100 μM), DOX (1.5 μM), or their combination. EGCG treatment was associated with S-phase accumulation, whereas DOX treatment resulted in G2/M accumulation. Combined EGCG + DOX treatment produced a broader redistribution of the cell-cycle profile, accompanied by an increased sub-G1 fraction. (**B**) Quantitative analysis of the G0/G1, S, G2/M, and sub-G1 fractions. Data are presented as mean ± SD from three independent biological experiments (*n* = 3). Statistical significance was evaluated using one-way ANOVA followed by Tukey’s multiple-comparisons test. * *p* < 0.05, ** *p* < 0.01, and *** *p* < 0.001 versus the untreated control.

Cell-cycle distribution was evaluated by PI staining using TALI image-based cytometry after 48 h of treatment. In untreated control cells, 61.4 ± 2.8% of the population was in G0/G1, 18.2 ± 1.5% in S phase, 18.4 ± 1.7% in G2/M, and 2.0 ± 0.8% in the sub-G1 fraction (Figure 7).

EGCG treatment markedly altered cell-cycle distribution, decreasing the G0/G1 population to 45.8 ± 2.1% (*p* < 0.01) and increasing the S-phase fraction to 31.6 ± 2.4% (*p* < 0.01), consistent with S-phase accumulation. DOX treatment produced a distinct pattern characterized by a reduction in the G0/G1 population to 38.5 ± 2.4% (*p* < 0.001) and an increase in the G2/M fraction to 41.2 ± 3.2% (*p* < 0.001), consistent with G2/M accumulation.

Combined EGCG + DOX treatment resulted in a broader redistribution of the cell-cycle profile, with 28.6 ± 2.3% of cells in G0/G1 (*p* < 0.001), 24.8 ± 2.2% in S phase (*p* < 0.05), and 36.4 ± 2.9% in G2/M (*p* < 0.01) compared with the untreated control. Notably, the combination group exhibited the highest sub-G1 fraction, reaching 10.2 ± 1.6% (*p* < 0.001 vs. control), compared with 2.0 ± 0.8%, 3.1 ± 0.9%, and 4.2 ± 1.1% in the control, EGCG, and DOX groups, respectively. These findings indicate treatment-specific alterations in cell-cycle distribution, with S-phase accumulation following EGCG treatment, G2/M accumulation following DOX treatment, and a broader redistribution accompanied by an increased sub-G1 population following combined treatment (Figure 7).

### 3.9. TALI Cytometry Confirms Treatment-Associated Cell Death in HeLa Cells

**Figure 8:**
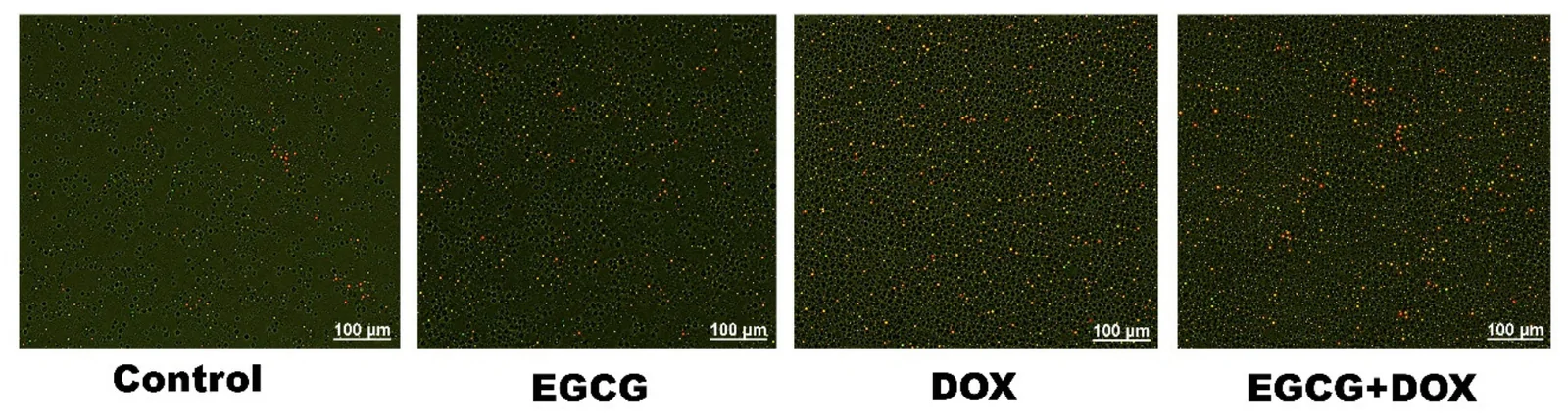
Representative fluorescence images of treatment-associated apoptotic cell death in HeLa cells using the Tali™ Apoptosis Kit (Annexin V Alexa Fluor^®^ 488/PI; Thermo Fisher Scientific, Waltham, MA, USA). HeLa cells were treated for 48 h with EGCG (100 μM), DOX (1.5 μM), or their combination (100 μM EGCG + 1.5 μM DOX) and analyzed using the Tali™ Image-Based Cytometer. Representative fluorescence images of the untreated control, EGCG, DOX, and EGCG + DOX groups are shown. Annexin V Alexa Fluor^®^ 488 positivity indicates phosphatidylserine externalization associated with apoptosis, whereas propidium iodide (PI) positivity indicates loss of plasma membrane integrity; Annexin V/PI double-positive cells are consistent with late apoptotic cells. Scale bars = 100 μm. Representative images from three independent biological experiments (*n* = 3) are shown.

TALI image-based cytometry was used to further evaluate treatment-associated changes in HeLa cell viability and cell death after 48 h of exposure to EGCG, DOX, or their combination. The viable cell population decreased from 80.0 ± 2.3% in the untreated control group to 70.2 ± 2.7% following EGCG treatment, 56.5 ± 2.8% following DOX treatment, and 39.0 ± 2.6% following combined EGCG + DOX treatment (Figure 8).

Concomitantly, the PI-positive dead-cell population increased from 18.0 ± 1.8% in the control group to 26.7 ± 2.1%, 39.3 ± 2.5%, and 50.8 ± 2.7% following EGCG, DOX, and EGCG + DOX treatment, respectively. The sub-G1 apoptotic fraction also increased from 2.0 ± 0.8% in the control group to 3.1 ± 0.9% with EGCG, 4.2 ± 1.1% with DOX, and 10.2 ± 1.6% with the combination. The highest dead-cell and sub-G1 fractions, together with the lowest viable-cell fraction, were therefore observed following combined EGCG + DOX treatment. These findings further support the treatment-associated increase in cell death observed in HeLa cells (Figure 8).

### 3.10. Treatment-Associated Changes in Intracellular DCF-Associated Fluorescence and NAC Modulation

**Figure 9:**
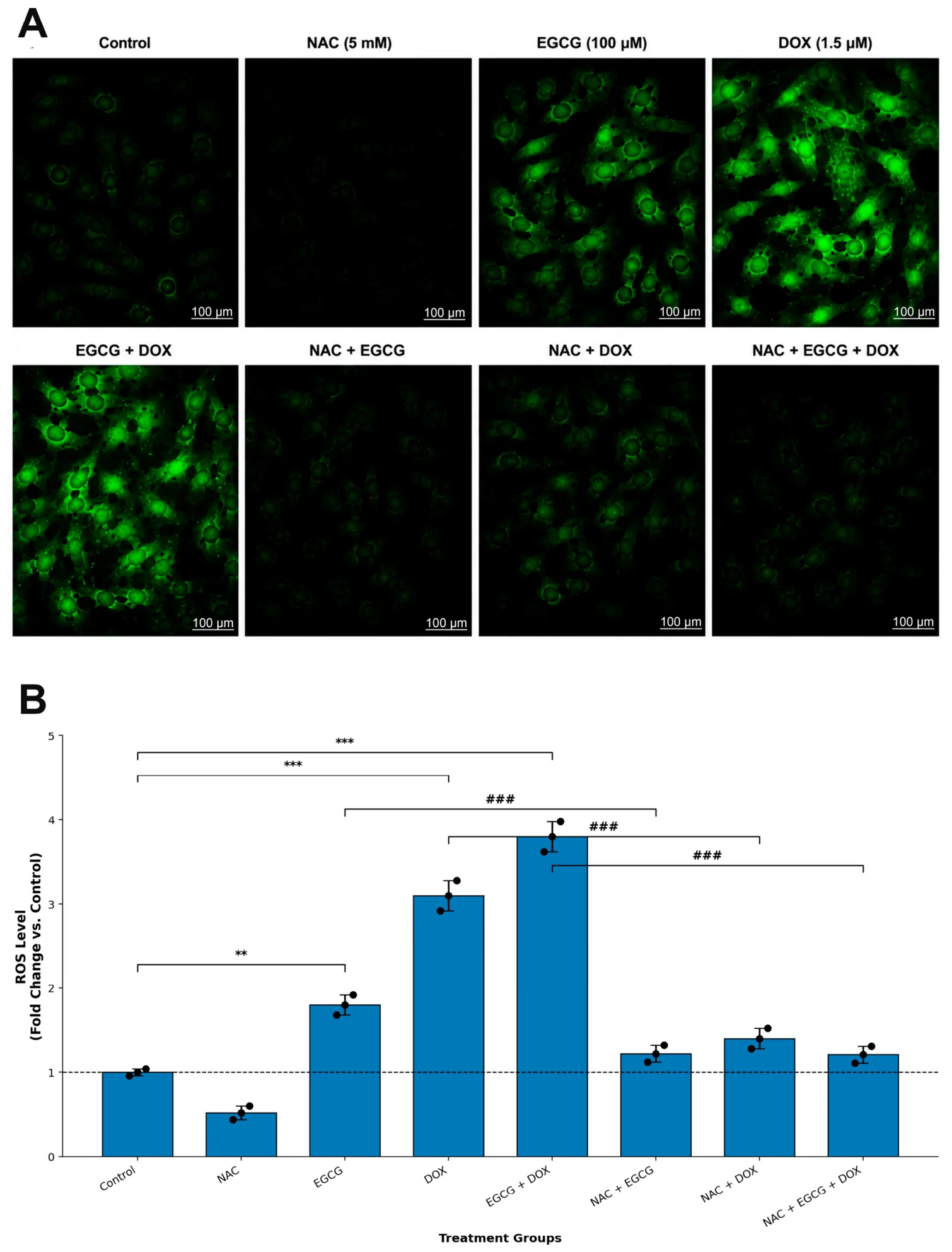
DCFH-DA fluorescence imaging and quantitative assessment of treatment-associated intracellular oxidative status following EGCG, DOX, and NAC exposure in HeLa cells. (**A**) Representative DCFH-DA fluorescence microscopy images of HeLa cells following 48 h of treatment with EGCG (100 μM), DOX (1.5 μM), or their combination, with or without NAC (5 mM; 2 h pretreatment). Increased green DCF-associated fluorescence was observed following EGCG and DOX exposure, with the strongest fluorescence observed in the EGCG + DOX group, whereas NAC pretreatment markedly attenuated treatment-associated fluorescence. Scale bars = 100 μm. (**B**) Quantitative analysis of intracellular DCFH-DA fluorescence. DCF-associated fluorescence values were normalized to the corresponding cell viability values obtained from parallel CCK-8 assays and expressed as fold change relative to the untreated control (1.0-fold). Individual data points represent three independent biological experiments (*n* = 3), and bars represent mean ± SD. Statistical comparisons were performed using one-way ANOVA followed by Tukey’s multiple-comparisons test. Asterisks indicate significant differences versus the untreated control (** *p* < 0.01, and *** *p* < 0.001), whereas hash symbols indicate significant differences between the corresponding treatment groups with and without NAC pretreatment (### *p* < 0.001). ns, not significant versus the untreated control.

Representative DCFH-DA fluorescence images demonstrated low basal DCF-associated fluorescence in untreated control cells, whereas the NAC-only group exhibited similarly low fluorescence intensity (Figure 9A). EGCG treatment produced a clear increase in intracellular DCF-associated fluorescence, while DOX treatment resulted in a more pronounced signal. The strongest fluorescence intensity was observed following combined EGCG + DOX treatment. In contrast, NAC pretreatment markedly attenuated the DCF-associated fluorescence observed following EGCG, DOX, and combined EGCG + DOX exposure. The NAC + EGCG and NAC + EGCG + DOX groups exhibited relatively weak fluorescence approaching basal levels, whereas modest residual fluorescence remained detectable in the NAC + DOX group.

Quantitative DCFH-DA analysis was consistent with the representative fluorescence images (Figure 9B). Relative to the untreated control (1.00-fold), DCF-associated fluorescence increased to 1.80-fold following EGCG treatment (*p* < 0.01), 3.10-fold following DOX treatment (*p* < 0.001), and 3.80-fold following combined EGCG + DOX treatment (*p* < 0.001). The highest DCF-associated fluorescence was therefore observed in the combination group. The NAC-only group showed lower basal fluorescence than the untreated control, consistent with the low fluorescence intensity observed in the representative images.

NAC pretreatment markedly attenuated the treatment-associated increases in DCF-associated fluorescence. Following NAC pretreatment, fluorescence levels were reduced to 1.22-fold in the NAC + EGCG group, 1.40-fold in the NAC + DOX group, and 1.21-fold in the NAC + EGCG + DOX group relative to the untreated control. DCF-associated fluorescence in the NAC + EGCG and NAC + EGCG + DOX groups was not significantly different from that of the untreated control, whereas a modest but statistically significant increase remained in the NAC + DOX group (1.40-fold, *p* < 0.05). NAC pretreatment also significantly reduced the fluorescence response relative to the corresponding treatments without NAC.

Collectively, the representative fluorescence images and quantitative measurements consistently demonstrated increased intracellular DCF-associated fluorescence following EGCG and DOX exposure, with the greatest increase observed following combined treatment. NAC pretreatment substantially attenuated these treatment-associated fluorescence responses, indicating that the observed DCFH-DA signal was sensitive to antioxidant pretreatment. These findings support a treatment-associated alteration in intracellular oxidative status but, in the absence of functional NAC rescue experiments assessing cell viability or apoptosis, do not establish oxidative stress as a causal mediator of EGCG- and DOX-induced cytotoxicity.

### 3.11. Treatment-Associated Changes in Caspase-3 Immunoreactivity

**Figure 10:**
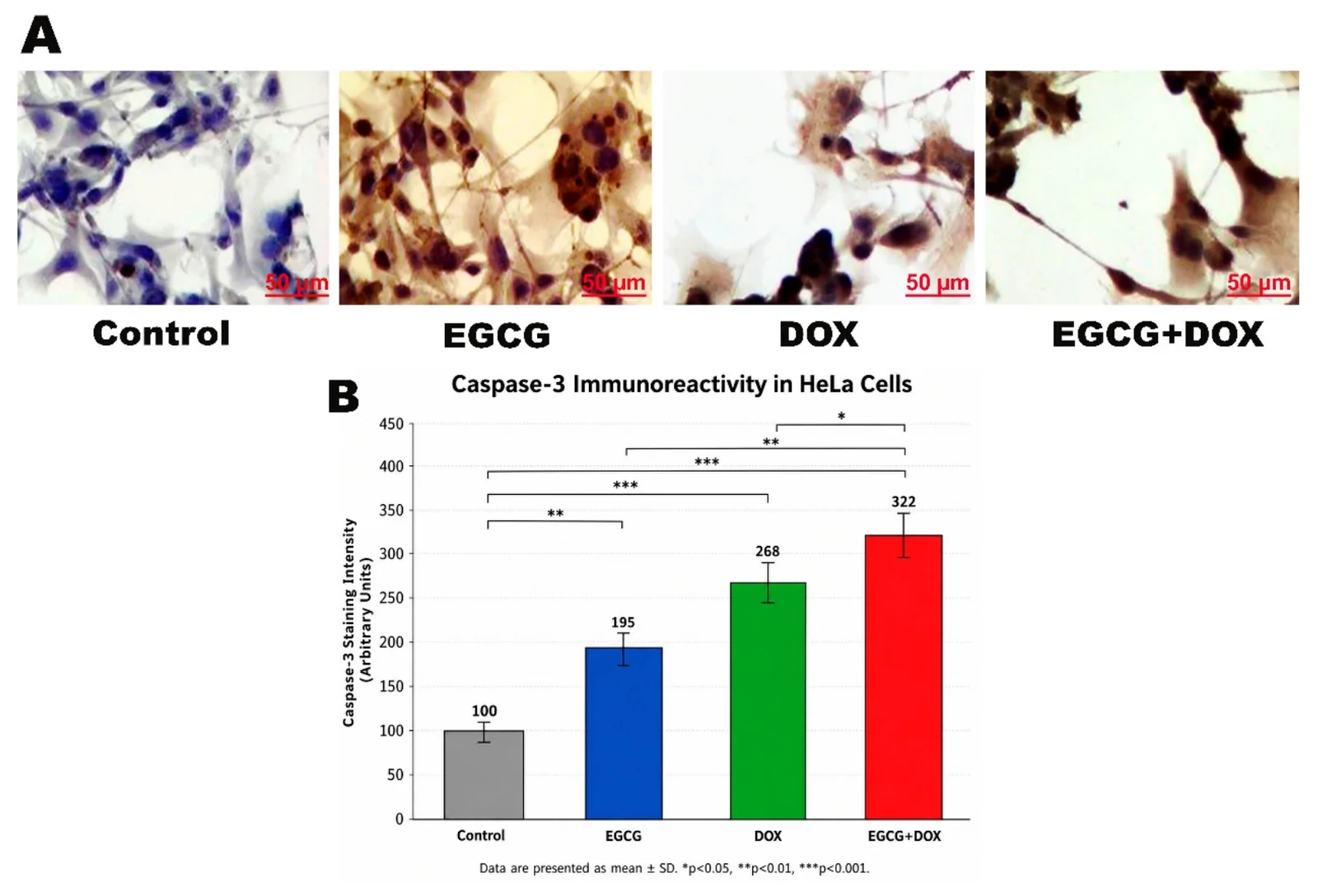
Caspase-3 immunocytochemical staining and semiquantitative analysis in HeLa cells following EGCG, DOX, and EGCG + DOX treatments. (**A**) Representative immunocytochemical micrographs showing total caspase-3 immunoreactivity visualized by DAB staining after 48 h of treatment with EGCG (100 μM), DOX (1.5 μM), or their combination (100 μM EGCG + 1.5 μM DOX), compared with the untreated control. Brown DAB staining represents caspase-3 immunoreactivity, whereas nuclei were counterstained with hematoxylin. Scale bars = 50 μm. (**B**) Semiquantitative image analysis of caspase-3 immunoreactivity expressed as DAB staining intensity in arbitrary units. Data are presented as mean ± SD from three independent biological experiments (*n* = 3). Statistical significance was evaluated using one-way ANOVA followed by Tukey’s multiple-comparisons test. * *p* < 0.05, ** *p* < 0.01, and *** *p* < 0.001.

Caspase-3 immunoreactivity was evaluated by DAB-based immunocytochemistry in HeLa cells after 48 h of treatment with EGCG (100 μM), DOX (1.5 μM), or their combination. Caspase-3-positive immunoreactivity was visualized as brown DAB staining, with hematoxylin used for nuclear counterstaining (Figure 10).

Untreated control cells exhibited relatively weak caspase-3 immunoreactivity, with a mean staining intensity of 100 ± 8 AU. EGCG treatment increased the staining intensity to 195 ± 14 AU (*p* < 0.01 vs. control), accompanied by more prominent cytoplasmic DAB immunoreactivity. A further increase was observed following DOX treatment, with a mean staining intensity of 268 ± 20 AU (*p* < 0.001 vs. control).

The highest caspase-3 immunoreactivity was observed following combined EGCG + DOX treatment, reaching 322 ± 28 AU (*p* < 0.001 vs. control). The staining intensity in the combination group was also significantly higher than that observed following EGCG (*p* < 0.01) or DOX (*p* < 0.05) treatment alone. Overall, these findings demonstrate a treatment-associated increase in total caspase-3 immunoreactivity, with the highest staining intensity observed in the EGCG + DOX group (Figure 10). Because the antibody used recognizes total rather than specifically cleaved caspase-3, these findings should not be interpreted as direct evidence of caspase-3 enzymatic activation.

### 3.12. Treatment-Associated Changes in Apoptosis- and Survival-Related Gene Expression

**Figure 11:**
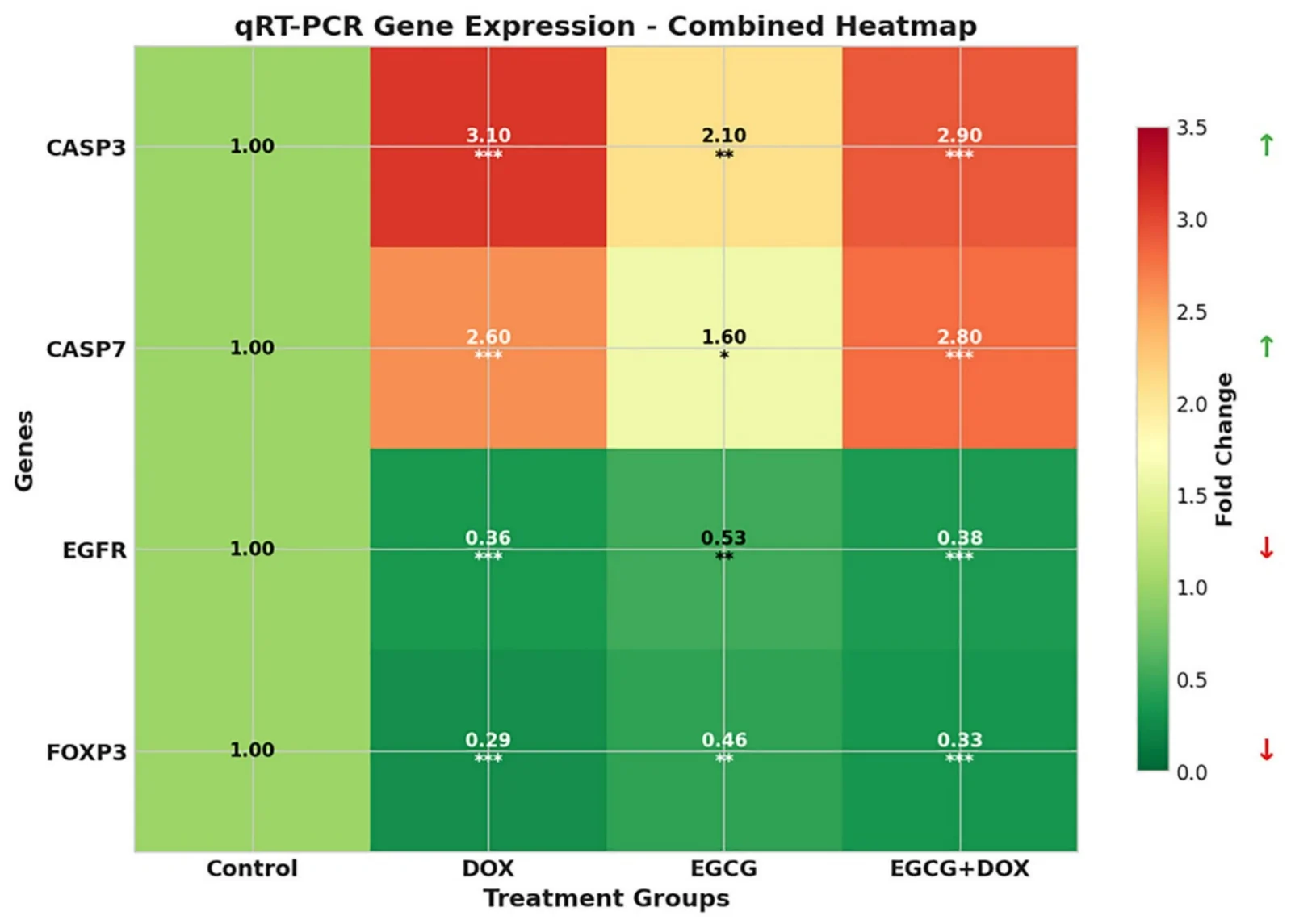
Relative mRNA expression of *EGFR*, *FOXP3*, *CASP3*, and *CASP7* in HeLa cells following 48 h of treatment with EGCG (100 µM), DOX (1.5 µM), or the EGCG + DOX combination (100 µM + 1.5 µM), as determined by RT-qPCR. Gene expression levels were normalized to *GAPDH* and expressed as fold change relative to the untreated control group (set to 1.0). Treatment with EGCG, DOX, and EGCG + DOX was associated with decreased *EGFR* and *FOXP3* mRNA expression and increased *CASP3* and *CASP7* mRNA expression relative to the control. The lowest mean *EGFR* and *FOXP3* expression levels and the highest mean *CASP3* expression were observed in the DOX-treated group, whereas the highest mean *CASP7* expression was observed in the combination group. Data are presented as mean ± SD from three independent biological experiments (*n* = 3). Statistical significance was determined by one-way ANOVA followed by Tukey’s multiple comparisons test (* *p* < 0.05, ** *p* < 0.01, *** *p* < 0.001 vs. control).

RT-qPCR analysis revealed treatment-associated changes in *EGFR*, *FOXP3*, *CASP3*, and *CASP7* mRNA expression in HeLa cells after 48 h of treatment (Figure 11).

Compared with the untreated control, *EGFR* mRNA expression decreased to 0.53 ± 0.05-fold following EGCG treatment (*p* < 0.01), 0.36 ± 0.06-fold following DOX treatment (*p* < 0.001), and 0.38 ± 0.04-fold following combined EGCG + DOX treatment (*p* < 0.001). The lowest mean *EGFR* expression was observed in the DOX-treated group.

Similarly, *FOXP3* mRNA expression decreased to 0.46 ± 0.06-fold following EGCG treatment (*p* < 0.01), 0.29 ± 0.05-fold following DOX treatment (*p* < 0.001), and 0.33 ± 0.04-fold following combined treatment (*p* < 0.001). The lowest mean *FOXP3* expression was observed following DOX treatment.

In contrast, *CASP3* mRNA expression increased to 2.10 ± 0.20-fold following EGCG treatment (*p* < 0.01), 3.10 ± 0.30-fold following DOX treatment (*p* < 0.001), and 2.90 ± 0.40-fold following combined EGCG + DOX treatment (*p* < 0.001). The highest mean *CASP3* expression was observed in the DOX-treated group.

*CASP7* mRNA expression increased to 1.60 ± 0.20-fold following EGCG treatment (*p* < 0.05), 2.60 ± 0.30-fold following DOX treatment (*p* < 0.001), and 2.80 ± 0.30-fold following combined treatment (*p* < 0.001). The highest mean *CASP7* expression was observed in the combination group. Overall, the treatments were associated with decreased *EGFR* and *FOXP3* mRNA expression and increased *CASP3* and *CASP7* mRNA expression relative to the untreated control (Figure 11). These findings represent treatment-associated transcriptional changes and should not be interpreted as evidence of corresponding changes in EGFR or FOXP3 protein abundance or functional signaling activity.

### 3.13. Predictive Functional Landscape of Treatment-Responsive Genes

To further contextualize the treatment-associated transcriptional changes observed in HeLa cells, predictive bioinformatic analyses were performed using the treatment-responsive genes evaluated by RT-qPCR (*EGFR*, *FOXP3*, *CASP3*, and *CASP7*) as the input gene set. PPI network analysis and GO and KEGG pathway enrichment analyses were used to examine potential functional relationships and signaling pathways associated with these transcriptional alterations. The resulting analyses provided a hypothesis-generating framework for interpreting the experimentally observed gene expression changes and were not considered independent experimental validation of the underlying molecular mechanisms.

#### 3.13.1. PPI Network Reveals Highly Connected Candidate Nodes

**Figure 12:**
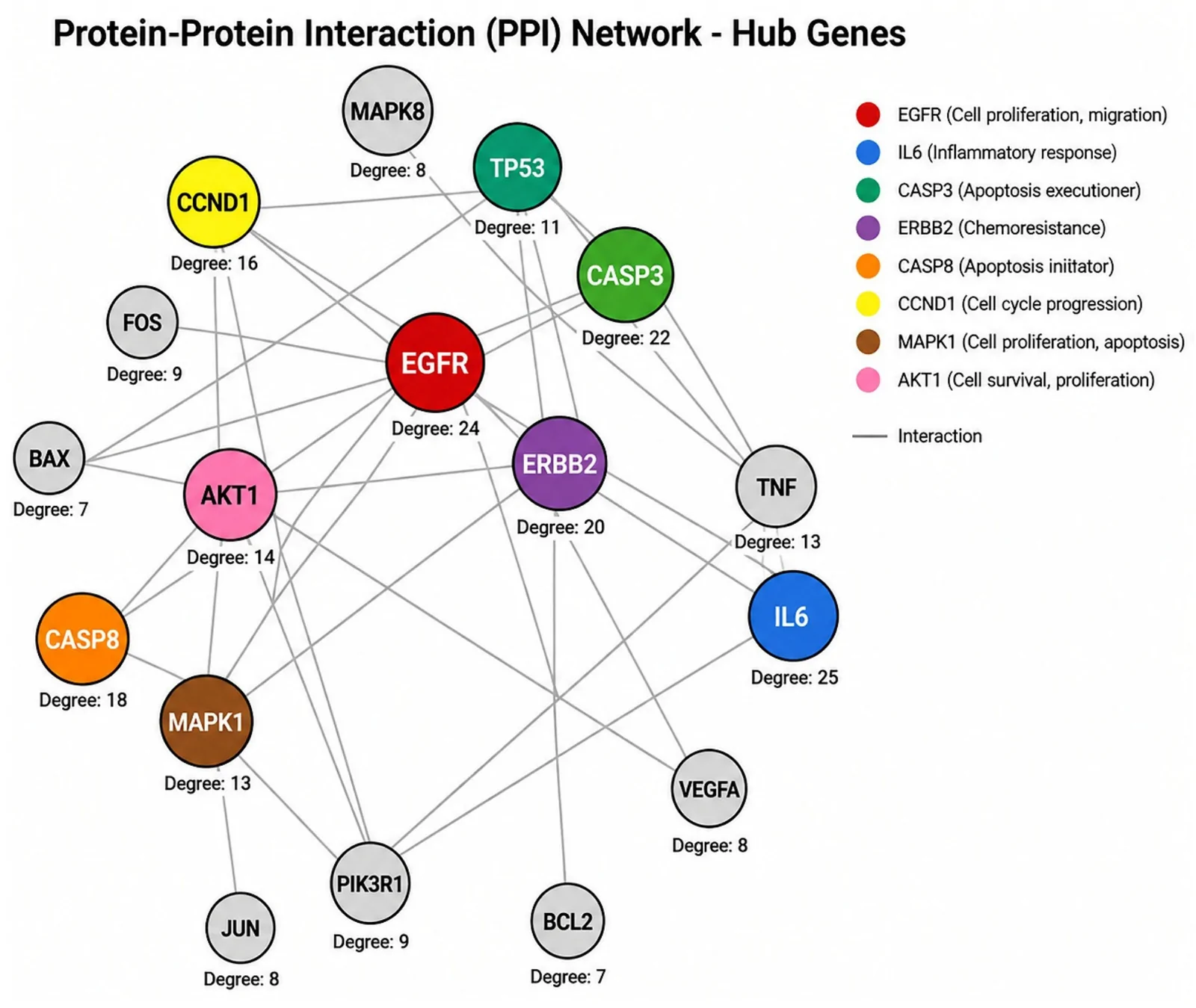
PPI network associated with the treatment-responsive genes identified by RT-qPCR and their functionally associated proteins. The interaction network was generated using the STRING database and visualized with Cytoscape. Node size reflects the connectivity measure used for network visualization. EGFR, IL6, CASP3, ERBB2, CASP8, CCND1, MAPK1, and AKT1 are highlighted as candidate proteins of interest within the interaction network, whereas gray nodes represent additional interacting proteins (TP53, TNF, MAPK8, VEGFA, JUN, PIK3R1, BAX, BCL2, and FOS). Edges represent known or predicted functional protein associations retrieved from STRING. The highlighted proteins should be interpreted as network-derived candidate nodes rather than experimentally validated hub proteins or treatment targets. The network represents a predictive, hypothesis-generating analysis and does not constitute experimental validation of the identified protein associations.

PPI network analysis was performed to explore potential functional relationships associated with the treatment-responsive genes identified by RT-qPCR. The STRING-derived interaction network was visualized using Cytoscape, and node connectivity was evaluated according to degree centrality (Figure 12). Among the proteins retained in the final interaction network, *IL6* exhibited the highest degree of connectivity (degree = 25), followed by *EGFR* (degree = 24), *CASP3* (degree = 22), *ERBB2* (degree = 20), *CASP8* (degree = 18), *CCND1* (degree = 16), *AKT1* (degree = 14), and *MAPK1* (degree = 13). Additional interacting proteins, including *TNF*, *TP53*, *MAPK8*, *BAX*, *FOS*, *PIK3R1*, *JUN*, *VEGFA*, and *BCL2*, were retained as secondary nodes within the network. Disconnected nodes were excluded from the final visualization according to the predefined network settings.

The connectivity pattern indicated that the experimentally identified transcriptional alterations were associated with a broader predicted interaction network involving proteins related to apoptosis, cell proliferation, survival, and inflammatory signaling. In particular, the high connectivity of *EGFR* and *CASP3*, both of which were experimentally evaluated by RT-qPCR, provided a network-level context for the observed transcriptional changes. However, the additional proteins identified through the PPI analysis represent predicted functional interactors and candidate nodes rather than experimentally validated treatment targets. Therefore, these network findings should be interpreted as hypothesis-generating associations requiring subsequent experimental validation.

#### 3.13.2. Functional Enrichment Across GO Categories

**Figure 13:**
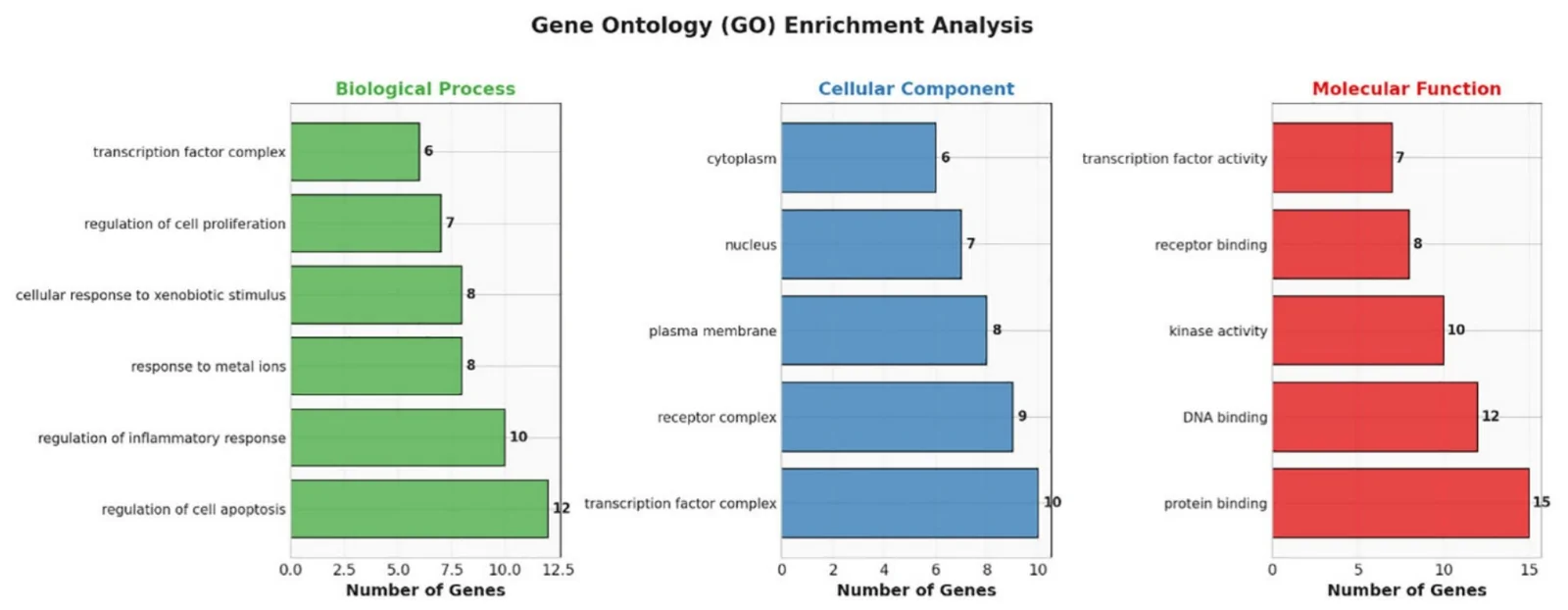
GO enrichment analysis of the predicted interaction network derived from the treatment-responsive genes evaluated by RT-qPCR and their functionally associated interacting proteins. Enriched GO terms are presented according to the BP, CC, and MF categories. In the BP category, the most represented terms included regulation of cell apoptosis, regulation of inflammatory response, response to metal ions, cellular response to xenobiotic stimulus, regulation of cell proliferation, and transcription factor complex. In the CC category, the represented terms included transcription factor complex, receptor complex, plasma membrane, nucleus, and cytoplasm. In the MF category, protein binding, DNA binding, kinase activity, receptor binding, and transcription factor activity were prominently represented. Bar lengths indicate the number of genes associated with each GO term within the analyzed network. The enrichment profile provides a predictive functional context for the network and should be interpreted as hypothesis-generating rather than as experimental evidence of direct pathway modulation.

GO enrichment analysis was performed to characterize the functional profile of the genes represented in the interaction network across the BP, CC, and MF categories (Figure 13).

Within the BP category, the most represented term was regulation of cell apoptosis (12 genes), followed by regulation of inflammatory response (10 genes). Response to metal ions and cellular response to xenobiotic stimulus each included eight genes, whereas regulation of cell proliferation included seven genes and transcription factor complex included six genes. These enriched terms indicated predicted functional associations predominantly related to apoptotic regulation, inflammatory responses, cellular responses to external stimuli, and proliferation.

Within the CC category, transcription factor complex showed the highest representation (10 genes), followed by receptor complex (9 genes), plasma membrane (8 genes), nucleus (7 genes), and cytoplasm (6 genes), indicating that the network-associated proteins were distributed across multiple cellular compartments and regulatory complexes.

Within the MF category, protein binding was the most highly represented term (15 genes), followed by DNA binding (12 genes), kinase activity (10 genes), receptor binding (8 genes), and transcription factor activity (7 genes). Collectively, these enrichment patterns suggest that the treatment-responsive transcriptional changes identified in the present study are associated with a broader predicted functional network involving apoptosis, inflammatory regulation, cellular proliferation, and diverse protein- and signaling-related functions. Given the predictive nature of the enrichment analysis, these associations should be considered hypothesis-generating rather than evidence of direct functional modulation by EGCG and DOX (Figure 13).

#### 3.13.3. KEGG Pathway Enrichment Highlights Multiple Signaling Networks

**Figure 14:**
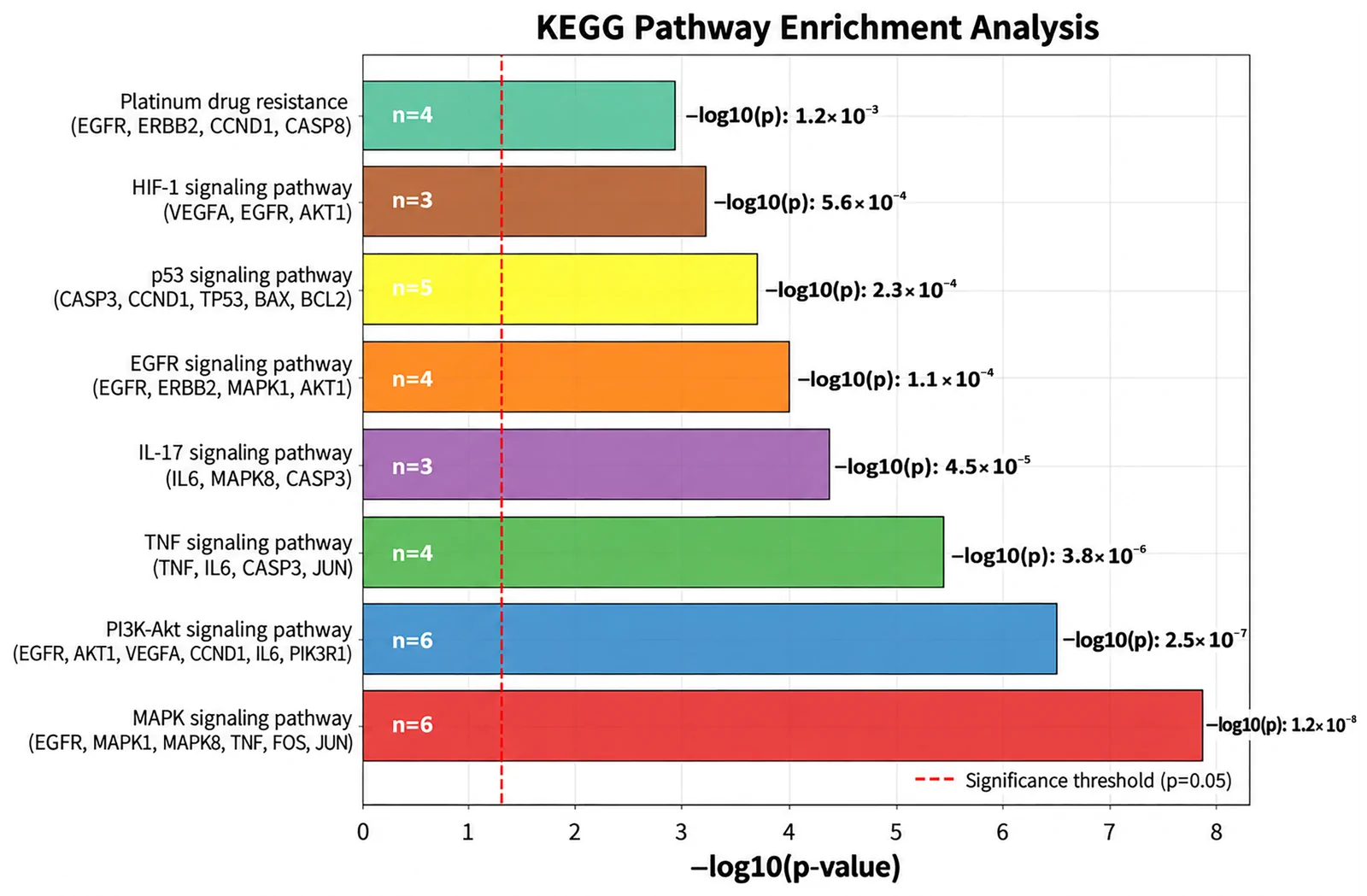
KEGG pathway enrichment analysis of the predicted interaction network derived from the differentially expressed genes identified by RT-qPCR and their functionally associated interacting proteins. Enrichment analysis identified signaling and regulatory pathways potentially associated with the observed transcriptional alterations following EGCG and DOX treatment. The enriched pathways are ranked according to −log10(*p*-value) and include the MAPK signaling pathway, PI3K–Akt signaling pathway, TNF signaling pathway, IL-17 signaling pathway, EGFR signaling pathway, p53 signaling pathway, HIF-1 signaling pathway, and platinum drug resistance. The numbers within the bars indicate the number of genes represented in the analyzed network that are associated with each pathway. The red dashed line indicates the significance threshold (*p* = 0.05).

KEGG pathway enrichment analysis identified several significantly enriched signaling and regulatory pathways within the predicted interaction network (Figure 14). Among the pathways shown, the MAPK signaling pathway exhibited the strongest statistical enrichment (*p* = 1.2 × 10^−8^; six genes: EGFR, MAPK1, MAPK8, TNF, FOS, and JUN), followed by the PI3K–Akt signaling pathway (*p* = 2.5 × 10^−7^; six genes: EGFR, AKT1, VEGFA, CCND1, IL6, and PIK3R1) and the TNF signaling pathway (*p* = 3.8 × 10^−6^; four genes: TNF, IL6, CASP3, and JUN). Significant enrichment was also observed for the IL-17 signaling pathway (*p* = 4.5 × 10^−5^; IL6, MAPK8, and CASP3), EGFR signaling pathway (*p* = 1.1 × 10^−4^; EGFR, ERBB2, MAPK1, and AKT1), p53 signaling pathway (*p* = 2.3 × 10^−4^; CASP3, CCND1, TP53, BAX, and BCL2), HIF-1 signaling pathway (*p* = 5.6 × 10^−4^; VEGFA, EGFR, and AKT1), and platinum drug resistance (*p* = 1.2 × 10^−3^; EGFR, ERBB2, CCND1, and CASP8).

Collectively, these enrichment patterns indicate that the treatment-responsive transcriptional changes identified in the present study are associated with a broader predicted molecular network involving pathways related to cell proliferation and survival, apoptosis, cell-cycle regulation, inflammatory signaling, and treatment-response mechanisms. Notably, these pathway associations were derived from the experimentally identified RT-qPCR genes together with their network-associated proteins and therefore do not demonstrate direct modulation of the corresponding pathways by EGCG or DOX. Accordingly, the KEGG findings should be regarded as hypothesis-generating predictions that provide functional context for the experimental observations rather than as experimental confirmation of specific molecular mechanisms (Figure 14).

## 4. Discussion

The present study demonstrates that combined EGCG and DOX exposure produces multiple treatment-associated cellular responses in HeLa cervical cancer cells, including reduced cell viability and wound closure, increased apoptotic cell death, altered cell-cycle distribution, increased intracellular DCF-associated fluorescence, and changes in apoptosis- and proliferation-related gene expression. Importantly, the reduction in CCK-8-derived cell viability was supported by Calcein-AM/PI dual-fluorescence staining, which showed a progressive decrease in Calcein-AM-positive viable cells and an increase in PI-positive membrane-compromised cells, with the greatest changes observed following combined EGCG + DOX treatment. This complementary fluorescence-based assessment strengthens the viability findings by demonstrating treatment-associated effects using cellular esterase activity and plasma membrane integrity as endpoints distinct from the metabolic readout of the CCK-8 assay. Furthermore, NAC pretreatment substantially attenuated the increases in DCF-associated fluorescence induced by EGCG, DOX, and their combination, indicating that these treatment-associated alterations in intracellular oxidative status were sensitive to antioxidant modulation. However, because functional rescue of cell viability or apoptosis by NAC was not evaluated, these findings do not establish oxidative stress as a causal mediator of the observed cytotoxic effects. Cytotoxicity assays showed slightly lower IC_50_ values in HeLa cells than in the non-malignant HaCaT comparator cells under the experimental conditions used. Furthermore, CI analysis revealed synergistic interactions in HeLa cells, whereas predominantly additive or antagonistic interactions were observed in HaCaT cells. These findings suggest differential cellular sensitivity to EGCG and DOX and support further investigation of EGCG as a potential adjunct to DOX-based treatment strategies. This concept is of particular interest because the therapeutic use of DOX is limited by dose-dependent systemic toxicity, particularly cardiotoxicity. Accordingly, identifying natural compounds capable of enhancing anticancer responses to conventional chemotherapeutic agents remains an important objective in cancer research. Recent reviews have highlighted EGCG as a promising dietary polyphenol for combination cancer therapy because of its capacity to influence multiple signaling and cellular regulatory processes [25,26].

The cytotoxicity analyses showed that both EGCG and DOX reduced cell viability in a concentration- and time-dependent manner. HeLa cells consistently exhibited lower IC_50_ values than HaCaT cells, and the calculated selectivity indices were greater than 1 for both agents at 24 and 48 h. Nevertheless, these values indicate only slight differential sensitivity and should not be interpreted as evidence of definitive tumor selectivity. Moreover, HaCaT cells are immortalized human keratinocytes rather than tissue-matched normal cervical epithelial cells. Thus, the observed differences indicate a slight differential response between HeLa and HaCaT cells under the present experimental conditions but require confirmation in more physiologically relevant normal cervical epithelial models. Similar differential responses to EGCG have been described in cervical, breast, ovarian, and colorectal cancer models, in which malignant cells may exhibit greater susceptibility than non-malignant cells [27,28]. Differences in cellular redox homeostasis, metabolic activity, mitochondrial function, and antioxidant capacity have been proposed to contribute to such differential responses. Cancer cells frequently maintain elevated basal oxidative stress, potentially making them more vulnerable to additional redox perturbations induced by pharmacological treatment. The EGCG concentration used in the subsequent cellular and molecular experiments (100 μM) was selected on the basis of the experimentally determined 48 h concentration–response profile and was close to the IC_50_ region in HeLa cells. This concentration was therefore intended to provide an experimentally informative in vitro exposure rather than to reproduce clinically achievable systemic EGCG levels. Importantly, the pharmacokinetic behavior and bioavailability of EGCG may substantially limit systemic exposure in vivo, and the present findings obtained at 100 μM should not be directly extrapolated to physiological or clinically achievable EGCG concentrations. Future studies evaluating lower EGCG concentrations, including exposure ranges more relevant to in vivo conditions, will be necessary to determine whether the observed interaction with DOX is maintained at more clinically relevant exposures.

A notable finding of the present study was the differential interaction between EGCG and DOX in HeLa and HaCaT cells. Chou–Talalay analysis demonstrated synergistic pharmacological interactions in HeLa cells under the tested relative-dose combination schemes. In contrast, HaCaT cells predominantly exhibited antagonistic or additive interactions. These findings indicate that the pharmacological interaction between EGCG and DOX was cell-context dependent, with synergism preferentially observed in HeLa cells under the conditions examined. Previous studies have suggested that EGCG can influence the activity of conventional chemotherapeutic agents through mechanisms involving PI3K/AKT and NF-κB signaling, multidrug resistance-associated processes, and mitochondrial apoptotic pathways [29,30]. However, these specific mechanisms were not directly examined in the present study. Therefore, the observed CI profile should primarily be interpreted as evidence of a pharmacological interaction at the cell-viability level rather than as direct demonstration of molecular chemosensitization.

Apoptotic cell death represented an important component of the treatment response. Annexin V-FITC/PI analysis demonstrated increased early and late apoptotic populations following EGCG and DOX treatment, with the combination group exhibiting the greatest apoptotic response and a corresponding reduction in the viable cell population. The complementary Tali™ image-based analysis showed a consistent treatment-associated shift toward apoptotic and membrane-compromised cell populations. Together, these independent observations support apoptosis as an important component of the cellular response to combined EGCG and DOX exposure. Previous studies have associated EGCG-induced apoptosis with mitochondrial dysfunction, cytochrome c release, and regulation of caspase-dependent pathways [31]. In the present study, the increased *CASP3* and *CASP7* mRNA expression and enhanced total caspase-3 immunoreactivity provide additional evidence consistent with involvement of apoptosis-related pathways. However, because cleavage or enzymatic activation of caspase-3 and caspase-7 was not directly measured, these findings should not be interpreted as direct evidence of caspase activation.

Cell-cycle analysis further demonstrated distinct treatment-associated alterations in HeLa cells. EGCG increased the S-phase population, whereas DOX markedly increased the G2/M fraction, consistent with the established effects of DOX on topoisomerase II-associated DNA damage and cell-cycle progression. The combination treatment produced redistribution across both S and G2/M phases rather than a greater G2/M accumulation than DOX alone. Importantly, the combination group exhibited the highest sub-G1 fraction, consistent with the enhanced apoptotic response observed in the Annexin V-based analyses. Previous studies have similarly reported that EGCG can interfere with DNA synthesis and cell-cycle regulatory processes and may modify cellular responses to DNA-damaging chemotherapeutic agents [30,32]. Nevertheless, cyclins, cyclin-dependent kinases, checkpoint proteins, and DNA damage markers were not directly evaluated in the present study. The observed changes should therefore be interpreted as treatment-associated redistribution of cell-cycle populations rather than evidence for a specific checkpoint mechanism.

Intracellular oxidative stress was also markedly altered by treatment. The higher signal in the combination group indicates a greater intracellular oxidative response under combined treatment conditions. EGCG is widely recognized for antioxidant properties under many physiological conditions; however, context-dependent pro-oxidant effects have also been described, particularly in cancer cells, where EGCG may influence cellular redox homeostasis and promote hydrogen peroxide generation. Excessive oxidative stress can affect mitochondrial integrity and apoptosis-associated signaling processes [33,34]. Consistent with a redox-sensitive component of the observed response, NAC pretreatment markedly attenuated the DCFH-DA signal, reducing it toward control levels in all treatment groups. However, the NAC experiments in the present study assessed ROS levels only and did not evaluate whether NAC rescued cell viability or reduced apoptosis. Consequently, these findings demonstrate that the treatment-associated DCFH-DA signal was sensitive to antioxidant pretreatment but do not establish that ROS was causally responsible for the cytotoxic or apoptotic effects of EGCG and DOX. Functional rescue experiments combining NAC pretreatment with viability and apoptosis measurements will be required to determine the extent to which oxidative stress contributes to the observed biological responses.

The transcriptional findings provided additional molecular context for these cellular responses. Treatment was associated with decreased *EGFR* and *FOXP3* mRNA expression and increased *CASP3* and *CASP7* mRNA expression. Notably, DOX produced the lowest mean *EGFR* and *FOXP3* mRNA expression and the highest mean *CASP3* mRNA expression, whereas the combination group exhibited the highest mean *CASP7* mRNA expression. EGFR is an important regulator of proliferation, survival, migration, and therapeutic responses in several malignancies, including cervical cancer, through signaling networks that include PI3K/AKT and MAPK pathways [35,36]. The observed decrease in *EGFR* mRNA expression is therefore biologically consistent with the phenotypic changes detected experimentally; however, the present RT-qPCR data do not establish a corresponding reduction in EGFR protein abundance or functional inhibition of EGFR-associated signaling. Accordingly, a causal relationship between the observed transcriptional change in *EGFR* and these phenotypic effects cannot be established from the present data. Similarly, increased *CASP3* and *CASP7* mRNA expression represents an apoptosis-associated transcriptional response that is consistent with the increased apoptotic cell populations observed after treatment [37]. The immunocytochemical increase in total caspase-3 immunoreactivity provides additional evidence of treatment-associated changes in caspase-3 abundance. However, the antibody used recognizes total rather than specifically cleaved caspase-3, and caspase enzymatic activity was not directly measured. Therefore, the present findings should not be interpreted as direct evidence of caspase-3 or caspase-7 activation. Regarding *FOXP3*, the observed decrease in mRNA expression represents an additional treatment-associated transcriptional alteration; however, FOXP3 protein abundance was not evaluated, and the functional significance of this transcriptional change in the present HeLa model remains to be established.

The predictive bioinformatic analyses provided a broader functional context for the experimentally observed transcriptional alterations. PPI network analysis identified EGFR as the most highly connected node, followed by IL6, CASP3, ERBB2, CASP8, CCND1, MAPK1, and AKT1 among the principal candidate hub proteins. GO enrichment associated the analyzed network with functional categories involving apoptosis, inflammatory regulation, cell proliferation, responses to external stimuli, protein binding, DNA binding, and kinase activity. KEGG analysis further identified significant enrichment of the MAPK, PI3K–Akt, TNF, IL-17, EGFR, p53, and HIF-1 signaling pathways, together with platinum drug resistance. These pathways have established roles in cellular proliferation, survival, inflammatory signaling, apoptosis, and treatment responses [38,39]. Importantly, most network-associated proteins were not experimentally measured in the present study, and enrichment analysis does not demonstrate that these pathways were activated or inhibited by EGCG or DOX. The bioinformatic findings should therefore be regarded as hypothesis-generating predictions that provide a framework for future mechanistic studies rather than as independent confirmation of the experimentally observed effects.

The wound-healing assay demonstrated reduced wound closure following EGCG and DOX treatment, with the greatest reduction observed in the combination group. However, because the assay was performed at cytotoxic treatment concentrations, reduced wound closure cannot be attributed specifically to inhibition of cell migration. Treatment-associated reductions in cell number and proliferation, together with increased cell death, may also have contributed to the observed effect. Therefore, these findings should be interpreted as evidence of reduced wound closure under the tested conditions rather than as a demonstration of a specific antimigratory effect. Future experiments using subcytotoxic concentrations and migration-specific approaches, such as transwell assays, will be required to distinguish direct effects on migration from effects secondary to reduced proliferation, viability, or increased cell death.

Several limitations of the present study should be acknowledged. First, the experimental analyses were performed using a single cervical cancer cell line, HeLa. Therefore, the observed EGCG–DOX synergistic interaction and associated cellular and transcriptional responses should be considered specific to the HeLa model under the present experimental conditions and should not be generalized to cervical cancer as a whole. Validation in additional cervical cancer cell lines with different HPV status and molecular backgrounds, as well as appropriate in vivo models, will be required to determine the broader reproducibility and translational relevance of these findings. In addition, the 100 μM EGCG concentration used for the subsequent cellular and molecular experiments was selected from the experimentally determined in vitro concentration–response profile and should not be considered representative of clinically achievable systemic EGCG exposure. Therefore, the present findings cannot be directly extrapolated to clinically relevant EGCG concentrations, and confirmation at lower, physiologically and pharmacologically relevant exposure levels is required. Second, HaCaT cells were used as a non-malignant comparator; however, HaCaT cells are immortalized human keratinocytes and do not constitute a tissue-matched normal cervical epithelial model. Consequently, the observed differences between HeLa and HaCaT cells should be interpreted as differential cellular sensitivity rather than definitive cancer-specific selectivity. Third, although CCK-8 is a metabolism-dependent viability assay and may not alone fully distinguish reduced metabolic activity from loss of cell viability, the present study incorporated Calcein-AM/PI dual-fluorescence staining as an orthogonal assessment based on intracellular esterase activity and plasma membrane integrity. Nevertheless, this complementary analysis was performed only in HeLa cells at the selected 48 h treatment condition and therefore does not provide an independent fluorescence-based validation of the full concentration–response profiles or the differential responses observed between HeLa and HaCaT cells. Fourth, although RT-qPCR demonstrated treatment-associated alterations in *EGFR*, *FOXP3*, *CASP3*, and *CASP7* expression, protein-level assessment was limited to total caspase-3 immunoreactivity by immunocytochemistry. Protein-level confirmation of EGFR, FOXP3, and CASP7, as well as evaluation of cleaved caspase-3 and caspase-7, would strengthen the mechanistic interpretation. Therefore, the observed *EGFR* and *FOXP3* mRNA changes should not be interpreted as evidence of corresponding changes in protein abundance or functional pathway activity. Moreover, caspase enzymatic activity was not directly assessed; therefore, the observed *CASP3* and *CASP7* transcriptional changes and total caspase-3 immunoreactivity cannot establish caspase activation. Fifth, NAC pretreatment experiments were limited to measurement of intracellular DCFH-DA fluorescence; functional rescue of cell viability or apoptosis was not evaluated, precluding conclusions regarding a causal requirement for ROS in treatment-induced cell death. Sixth, DCFH-DA provides a general indicator of intracellular oxidation and does not identify individual ROS species or their specific intracellular sources. In addition, an independent positive control for probe oxidation, such as H_2_O_2_ exposure, was not included; therefore, the sensitivity and dynamic range of the DCFH-DA assay were not independently verified under the present experimental conditions. Accordingly, the DCFH-DA/NAC findings should be interpreted as evidence of treatment-associated and antioxidant-sensitive alterations in intracellular oxidative status rather than as definitive evidence that ROS causally mediate the cytotoxic response. Seventh, the wound-healing assay was performed at cytotoxic treatment concentrations, and therefore an independent migration-specific effect cannot be fully separated from changes in cell number, proliferation, viability, and treatment-induced cell death. Accordingly, the observed reduction should be interpreted as reduced wound closure rather than as definitive evidence of an antimigratory effect. Eighth, the PPI, GO, and KEGG analyses were derived from a limited experimentally identified gene set expanded through database-defined functional interactions; consequently, the resulting candidate proteins and pathways require independent experimental validation. Finally, other potentially relevant mechanisms, including DNA damage responses, autophagy, mitochondrial dysfunction, and metabolic reprogramming, were not directly investigated and warrant examination in future studies.

## 5. Conclusions

The present study demonstrates that EGCG modifies the cellular response to DOX in HeLa cervical cancer cells under the tested in vitro conditions. EGCG–DOX combinations produced synergistic pharmacological interactions in HeLa cells across the tested relative-dose combination schemes, whereas comparable synergism was not observed in the HaCaT comparator cells. Combined treatment was associated with reduced cell viability and wound closure, increased apoptosis, altered cell-cycle distribution, antioxidant-sensitive changes in intracellular DCF-associated fluorescence, and treatment-associated alterations in *EGFR*, *FOXP3*, *CASP3*, and *CASP7* mRNA expression. These findings do not establish cancer-specific selectivity, ROS-dependent cytotoxicity, caspase activation, or a definitive molecular mechanism. Overall, the results support a cell-context-dependent interaction between EGCG and DOX and provide a basis for further investigation in additional cervical cancer and tissue-matched non-malignant models, at clinically relevant exposures, and in vivo before the therapeutic relevance of this combination can be established.

## Figures and Tables

**Table 1:** Primer sequences.

Genes	Forward Primer (5′–3′)	Reverse Primer (5′–3′)
***EGFR***	AACACCCTGGTCTGGAAGTACG	TCGTTGGACAGCCTTCAAGACC
***FOXP3***	GGCACAATGTCTCCTCCAGAGA	CAGATGAAGCCTTGGTCAGTGC
***CASP7***	CGGAACAGACAAAGATGCCGAG	AGGCGGCATTTGTATGGTCCTC
***CASP3***	GGAAGCGAATCAATGGACTCTGG	GCATCGACATCTGTACCAGACC
***GAPDH***	GGAGCGAGATCCCTCCAAAAT	GGCTGTTGTCATACTTCTCATGG

**Table 2:** Cell viability following combined EGCG and DOX exposure under the 1:2 relative-dose scheme in HeLa and HaCaT cells.

EGCG + DOX (μM)	HeLa Cell Viability (%)	HaCaT Cell Viability (%)
12.5 + 0.25	43.0	87.0
25 + 0.50	29.0	79.0
50 + 1.00	18.0	71.0
100 + 2.00	12.0	61.0
200 + 4.00	8.0	52.0

Cell viability was determined after 48 h of combined EGCG and DOX exposure using the CCK-8 assay and is expressed relative to the untreated control. Data were obtained from three independent biological experiments (*n* = 3). The concentration pairs correspond to the 1:2 relative-dose combination scheme defined in Section 2.3.

## Data Availability

The original contributions presented in this study are included in the article. Further inquiries can be directed to the corresponding author.

## Abbreviations

AKT	protein kinase B
ANOVA	analysis of variance
BAX	BCL2-associated X protein
BCL2	B-cell lymphoma 2
BP	Biological Process
BSA	bovine serum albumin
CASP3	caspase-3
CASP7	caspase-7
CASP8	caspase-8
CC	Cellular Component
CCK-8	Cell Counting Kit-8
CCND1	cyclin D1
CDK	cyclin-dependent kinase
CI	combination index
DAB	3,3′-diaminobenzidine
DCFH-DA	2′,7′-dichlorofluorescein diacetate
DMEM	Dulbecco’s Modified Eagle Medium
DOX	doxorubicin
EGCG	epigallocatechin gallate
EGFR	epidermal growth factor receptor
ERBB2	Erb-B2 receptor tyrosine kinase 2
FBS	fetal bovine serum
FDR	false discovery rate
FOXP3	forkhead box P3
GO	Gene Ontology
GPx	glutathione peroxidase
HIF-1	hypoxia-inducible factor 1
HPV	human papillomavirus
HRP	horseradish peroxidase
IC_50_	half-maximal inhibitory concentration
IL-6	interleukin-6
IL-17	interleukin-17
KEGG	Kyoto Encyclopedia of Genes and Genomes
LMP	lysosomal membrane permeabilization
MAPK	mitogen-activated protein kinase
MDR	multidrug resistance
MF	Molecular Function
NAC	N-acetyl-L-cysteine
NF-κB	nuclear factor kappa B
PBS	phosphate-buffered saline
PI	propidium iodide
PI3K	phosphoinositide 3-kinase
PPI	protein–protein interaction
ROS	reactive oxygen species
RT-qPCR	quantitative reverse transcription polymerase chain reaction
SD	standard deviation
SI	selectivity index
SOD	superoxide dismutase
TNF	tumor necrosis factor
Trx	thioredoxin
TrxR	thioredoxin reductase
VEGF	vascular endothelial growth factor
